# Mass Spectrometry-Based *De Novo* Sequencing of N-Glycosylated Light Chains: Linking Urinary Proteoforms to *In Situ* Renal Amyloid Deposits

**DOI:** 10.1016/j.mcpro.2026.101652

**Published:** 2026-09-01

**Authors:** Yueyue Zhu, Xin Wang, Xinyue Zhou, Shuang Wang, Peifeng Ji, Zidi Yan, Kai Chen, Xiaojuan Yu, Gang Liu, Li Yang, Yi Liu, Suxia Wang

**Affiliations:** 1 Laboratory of Electron Microscopy, Pathological Center, Peking University First Hospital, Beijing, People's Republic of China; 2 Renal Division, Department of Medicine, Peking University First Hospital, Beijing, People's Republic of China; 3 Renal Pathological Center, Institute of Nephrology, Peking University, Beijing, People's Republic of China; 4 Key Laboratory of Renal Disease, Ministry of Health of China, Beijing, People's Republic of China; 5 Key Laboratory of CKD Prevention and Treatment, Ministry of Education of China, Beijing, People's Republic of China; 6 Peking-Tsinghua Center for Life Sciences, Beijing, People's Republic of China; 7 College of Chemistry and Molecular Engineering, Peking University, Beijing, People's Republic of China; 8 Omics Technology Center, Peking University First Hospital, Beijing, People's Republic of China; 9 Research Center, Peking University First Hospital, Beijing, People's Republic of China; 10 Key Laboratory of Combinatorial Biosynthesis and Drug Discovery, Ministry of Education, School of Pharmaceutical Sciences, Wuhan University, Wuhan, People's Republic of China

**Keywords:** *de novo* sequencing, N-glycosylation, AL amyloidosis, free light chains, mass spectrometry

## Abstract

Systemic immunoglobulin light-chain (AL) amyloidosis is characterized by clonal immunoglobulin-secreting cells that produce a monoclonal light chain prone to misfolding and amyloid fibril formation in tissues. Understanding its molecular basis requires accurate full-length sequencing of amyloidogenic light chains and linkage of circulating light chains to renal deposits. Achieving this is technically challenging because the low abundance and N-glycosylation of amyloidogenic light chains can complicate protein purification and peptide-level sequence analysis. To address these challenges, we implemented a robust analytical pipeline that integrates intact-mass measurement by Q-TOF MS before and after deglycosylation with multi-protease digestion and *de novo* peptide sequencing to systematically characterize urinary N-glycosylated light chains. Mass shifts observed before and after deglycosylation supported the presence of N-glycosylation, whereas deglycosylated intact masses were used to constrain full-length sequence assembly. The assembled urinary light-chain sequences were subsequently compared with matched renal amyloid proteomes isolated by laser microdissection and analyzed by bottom-up liquid chromatography–tandem mass spectrometry (LC–MS/MS). Enzymatic deglycosylation reduced glycan-induced spectral interference. Eight monoclonal light-chain sequences were assembled from urinary light chains, including five derived from the immunoglobulin kappa variable 1 (*IGKV1*), two from the immunoglobulin lambda variable 2 (*IGLV2*), and one from *IGKV4*. The close agreement between theoretical and experimental intact masses confirmed the accuracy and completeness of sequence assembly. In each renal amyloid proteome, the corresponding urine-derived sequence had the highest score among light-chain identifications of the patient's clinically determined isotype (κ or λ) and showed 87.6% to 100% variable-region peptide coverage. Together, these findings support urinary N-glycosylated monoclonal light chains as the precursor proteins of the corresponding renal amyloid fibrils. In conclusion, we have demonstrated a robust workflow that applies established *de novo* peptide sequencing to characterize urinary N-glycosylated light chains and trace the corresponding sequences in renal amyloid deposits, with potential applications across light chain-related diseases.

## Highlights

• *De novo* sequencing and intact mass characterize amyloidogenic light chains.
• Urinary light-chain sequences show molecular concordance with *in situ* renal amyloid.
• This proteomic tracing workflow has potential in light chain-related diseases.

## Graphical Abstract

**Figure:**
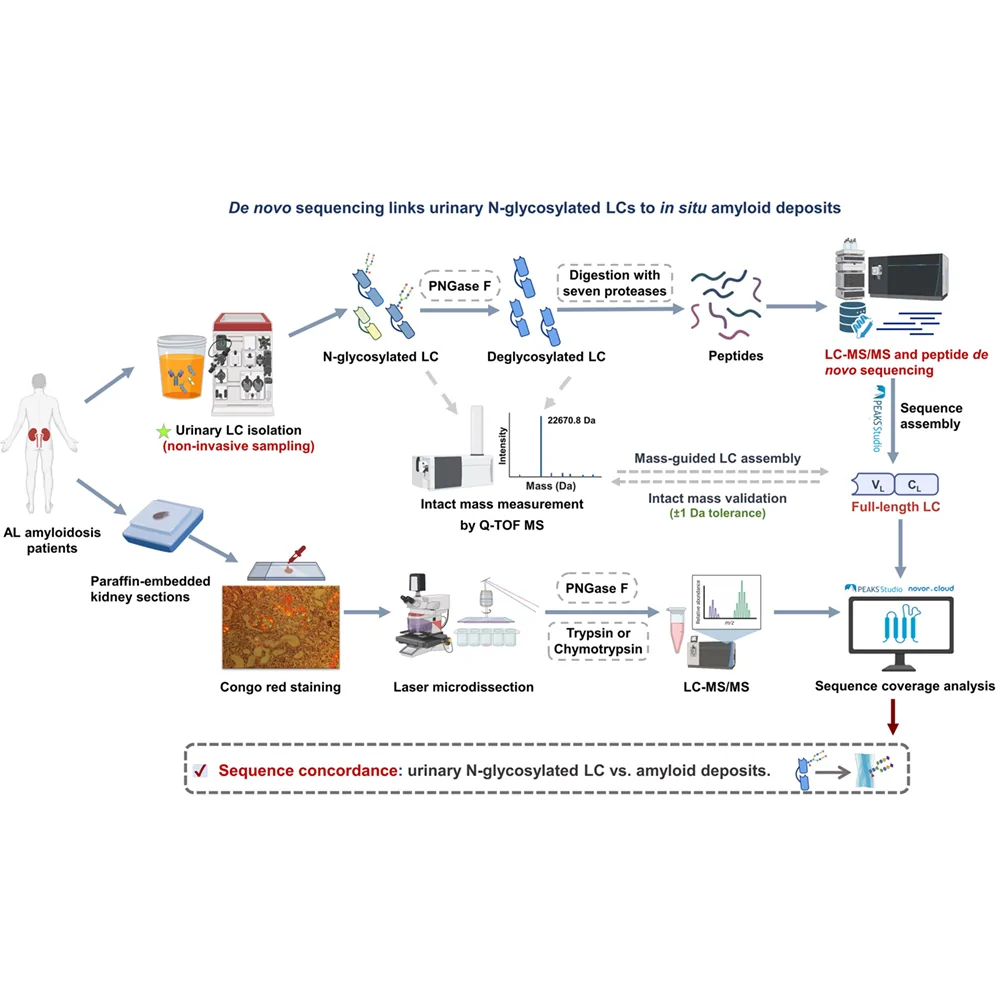

## In Brief

Here, we present an integrated workflow for sequencing urinary N-glycosylated monoclonal light chains in systemic immunoglobulin light-chain amyloidosis. The workflow combines molecular-mass measurements before and after deglycosylation with multiprotease digestion and established *de novo* peptide sequencing. Comparison with patient-matched laser-microdissected renal amyloid deposits demonstrated sequence agreement between urinary light chains and tissue amyloid fibrils. This workflow provides a non-invasive approach for acquiring amyloidogenic light-chain sequences and may facilitate molecular studies of light-chain–related diseases.

Systemic immunoglobulin light-chain (AL) amyloidosis is one of the most common forms of systemic amyloidosis and is characterized by clonal plasma cells that produce a monoclonal immunoglobulin light chain of either the kappa (κ) or the lambda (λ) type. In AL amyloidosis, disease-associated (amyloidogenic) light chains act as the precursor proteins of amyloid fibrils. Following misfolding, they assemble into extracellular amyloid fibrils that progressively damage affected organs (1). Light-chain sequence diversity arises from recombination of variable (V) and joining (J) gene segments, junctional diversity at the V–J junction, and somatic hypermutation, making amyloidogenic light-chain sequences highly patient specific (2). Structurally, a light chain consists of an N-terminal variable light-chain domain (V_L_) and a C-terminal constant light-chain domain (C_L_). The V_L_ contains three highly variable complementarity-determining regions (CDRs), which form the antigen-binding loops, separated by four relatively conserved framework regions (FRs) that maintain the immunoglobulin fold (3). The primary sequence of light chains is a major determinant of their amyloidogenicity in AL amyloidosis (4). Amyloidogenic light chains are often destabilized by mutations in the FRs or CDRs of the V_L_ domain; mutations in the C_L_ domain may also contribute. These critical amino acid substitutions may decrease thermodynamic stability, increase conformational dynamics, expose aggregation-prone regions, or alter interdomain interactions (5, 6).

In addition to sequence heterogeneity, post-translational modifications (PTMs), including N-terminal pyroglutamate (PyroGlu) formation and N-glycosylation, are frequently observed in AL amyloid fibrils (7, 8). Analyses of patient-derived monoclonal light chains indicate that N-glycosylated light chains are more frequently detected in AL amyloidosis than in other plasma cell dyscrasias (9, 10), occurring in approximately 14% to 33% of κ light chains and 2% to 10% of λ light chains (11). In patients with monoclonal gammopathy of undetermined significance, an asymptomatic monoclonal plasma cell dyscrasia that can precede AL amyloidosis, the presence of N-glycosylated monoclonal light chains has been associated with an increased risk of progression to AL amyloidosis (12, 13). Moreover, N-glycosylated AL amyloid fibrils were more resistant to proteinase K digestion than their deglycosylated counterparts (8). Therefore, determining the full-length amino acid sequences of these N-glycosylated light chains is crucial for further elucidating the pathogenic significance of N-glycosylation in AL amyloidosis.

Although highly sensitive rapid amplification of cDNA ends–based immunoglobulin repertoire sequencing (RACE-RepSeq) using bone marrow mRNA can identify clonal light-chain sequences (14), it requires invasive bone marrow aspiration and generally provides the V_L_ and partial C_L_ regions rather than the full-length light-chain sequence. Furthermore, *de novo* sequencing of tissue-derived amyloid fibrils is hindered by degradation of amyloidogenic light chains before deposition (15). In this context, *de novo* sequencing of urinary light chains is a noninvasive approach for obtaining full-length sequences in diseases with a high tumor burden (>10% bone marrow plasma cells), such as multiple myeloma (MM) and its associated kidney injury (16, 17). In contrast, isolated AL amyloidosis is characterized by a low clonal plasma cell burden and low circulating free light-chain levels, making it difficult to isolate highly pure monoclonal light chains from peripheral biofluid. Consequently, few studies have used serum- or urine-derived light chains for *de novo* sequencing in AL amyloidosis. Moreover, N-glycosylation further complicates this workflow, as labile glycan moieties interfere with peptide backbone fragmentation, generating complex spectra that hinder sequence interpretation (18).

In this study, we present an integrated workflow combining molecular-weight-constrained *de novo* sequence assembly with tissue proteomic validation. Enzymatic deglycosylation reduced glycan-induced spectral interference, while digestion with seven enzymes generated complementary peptides for sequence assembly. Molecular masses measured by Q-TOF mass spectrometry were used to constrain and validate the assembled full-length light-chain sequence. Proteomic analysis of laser-microdissected renal amyloid deposits further identified the urine-derived N-glycosylated light-chain sequences in the corresponding tissue deposits. This workflow enables non-invasive acquisition of full-length light-chain sequences in AL amyloidosis. More broadly, integrating sequencing of urine-derived monoclonal light chains with proteomic analysis of renal deposits may be extended to other light-chain–related diseases to link circulating light chains with their deposits in involved organs.

## Experimental Procedures

### Patient Cohort and Ethics Statement

Eight patients with renal biopsy-proven AL amyloidosis were enrolled, including six κ-type and two λ-type cases. All renal biopsy specimens were evaluated by routine light microscopy, direct immunofluorescence, and electron microscopy. The amyloid-associated light-chain isotype was clinically determined by direct immunofluorescence staining for κ and λ, with λ-type deposition in patient PtC1-λ further confirmed by immunoelectron microscopy. The median age at diagnosis was 58.5 years (range, 38–90 years). According to the 2014 renal prognostic staging system (19), five of the eight patients were classified as renal stage II. The median difference between involved and uninvolved free light chains (dFLC) was 160.8 mg/L (range, 65.6–647.2 mg/L) (Supplemental Table S1). This study included patients with AL amyloidosis who provided written informed consent at Peking University First Hospital. All procedures complied with the Declaration of Helsinki and were approved by the Ethics Committee of Peking University First Hospital (No. 2022-448).

### Experimental Design and Statistical Rationale

The study included eight patients with renal biopsy-proven AL amyloidosis, each representing an independent biological sample. For each patient, purified urinary light chains were divided into seven aliquots and subjected to complementary enzymatic digestion strategies to maximize peptide sequence coverage for full-length *de novo* sequence assembly. Corresponding laser-microdissected Congo red-positive renal amyloid deposits were analyzed following both tryptic and chymotryptic digestion to provide complementary sequence information for tissue proteomic validation. One liquid chromatography–tandem mass spectrometry (LC–MS/MS) acquisition was performed for each digest under each protease condition used for that sample.

This study was designed to reconstruct amyloidogenic light chains and assess their molecular concordance with renal amyloid deposits rather than perform quantitative proteomic comparisons. Accordingly, the primary study endpoints were sequence reconstruction, intact-mass agreement, peptide sequence coverage, and patient-matched urine–tissue sequence concordance. The reconstructed sequences were supported by agreement between experimental and theoretical intact masses following deglycosylation and complete peptide coverage obtained by database searching of the corresponding digested urinary light chains. Separately, tissue proteomics demonstrated extensive coverage of the patient-matched urine-derived V_L_ sequences in renal amyloid deposits.

### Urine Collection, Processing, and Light Chain Purification

Twenty-four–hour urine samples were collected after AL amyloidosis was confirmed by kidney biopsy. Each sample was collected in one or more 500-mL polystyrene urine collection containers without preservatives or protease inhibitors. Upon completion of the 24-h collection, the urine samples were concentrated at room temperature using a Pellicon 2 Mini Cassette equipped with a 10-kDa molecular-weight-cutoff membrane (Merck Millipore), transferred to 50-mL centrifuge tubes, and stored at −80 °C on the same day until further processing. Before affinity purification, thawed samples were centrifuged at 2500 rpm for 20 min at 4 °C and filtered through a 0.22 μm membrane (Merck Millipore). All samples were collected, processed, and stored using the same standardized workflow.

Free light chains were isolated on an ÄKTA pure system using HiTrap KappaSelect or LambdaFabSelect affinity columns (Cytiva), selected according to the AL subtype. Contaminating intact IgG was removed using a HiTrap Protein G HP column. The resulting light chains were further purified by size-exclusion chromatography on a Superdex 75 Increase 10/300 Gl column and underwent fewer than three freeze–thaw cycles before LC-MS/MS analysis.

### Molecular Mass Determination of Reduced Light Chains by Electrospray Ionization (ESI)–Q-TOF MS

Purified light chains (10 μg per condition) were divided into deglycosylated and nondeglycosylated groups. Deglycosylation was performed by incubating samples with PNGase F in 20 μl of 20 mM Tris-HCl (pH 8.0) at 37 °C overnight, while control samples were incubated in buffer alone. Following incubation, tris(2-carboxyethyl)phosphine (TCEP) was added to a final concentration of 25 mM, and samples were heated at 95 °C for 10 min to reduce disulfide bonds.

The molecular masses of the reduced full-length light chains were measured using a Waters Vion IMS QTof mass spectrometer coupled to an ACQUITY UPLC I-Class system. Proteins were separated on a PLRP-S column (1000 Å, 5 μm) at 0.2 ml/min with water containing 0.1% formic acid (FA) (mobile phase A) and acetonitrile containing 0.1% FA (mobile phase B). The liquid chromatography gradient started at 10% solvent B, was held for 1 min, increased linearly to 55% B at 3.5 min and to 95% B at 7 min, held at 95% B until 9 min, and then returned to 10% B for a 1 min re-equilibration (total run time, 10 min). Mass spectra were acquired in positive ESI mode (ESI+) over an m/z range of 500 to 2000, with a capillary voltage of 2.8 kV and a desolvation temperature of 280 °C. Molecular masses were obtained by charge-state deconvolution using UNIFI software (Waters).

### Calculation of Theoretical Molecular Weight

Theoretical average MWs were calculated from the assembled amino acid sequences using the ExPASy Compute pI/Mw tool (https://web.expasy.org/compute_pi/). For PNGase F-treated samples, each confirmed glycosylated Asn was replaced with Asp to account for the approximately +0.98 Da mass shift caused by enzymatic deglycosylation. For immunoglobulin lambda variable 2 to 14 (*IGLV2-14*)-derived sequences, an additional −17.03 Da adjustment was applied to account for the cyclization of N-terminal Gln to pyroGlu. The calculated molecular weights were compared with the deconvoluted masses of the reduced full-length light chains.

### Sample Preparation for *De Novo* Sequencing

Purified light chains (70 μg) were denatured with urea, reduced with dithiothreitol (DTT), and alkylated with iodoacetamide (IAA). N-linked glycans were subsequently removed by incubation with PNGase F in Tris-HCl buffer (pH 8.0). The deglycosylated sample was then split into equal portions and subjected to proteolytic cleavage using the following seven enzymes: trypsin, Asp-N, pepsin, chymotrypsin, Glu-C + trypsin, elastase, or *Nepenthes* digestive fluid. The six commercial proteases were used according to the manufacturer's instructions (Promega). *Nepenthes* digestion was carried out in 100 mM glycine-HCl buffer (pH 2.5) as described by Rey *et al*. (20). Because of its limited purity, the PtZ2-κ sample was subjected to in-gel digestion. The gel band corresponding to the N-glycosylated species was excised and subjected to in-gel reduction with DTT, alkylation with IAA, and overnight PNGase F treatment, as previously described (21, 22), followed by overnight digestion with the same panel of proteases described above. Peptides were extracted stepwise with acetonitrile/trifluoroacetic acid, desalted and concentrated using ZipTip C18 tips (Merck KGaA), and analyzed by LC–MS/MS.

### LC-MS/MS Analysis, *De Novo* Sequencing, and Sequence Assembly

Peptides were analyzed using a Vanquish Neo UHPLC system (Thermo Fisher Scientific, Waltham, MA) operating in nanoflow mode. Peptides were separated on an in-house packed reversed-phase capillary column (75 μm inner diameter × 25 cm) packed with 1.9 μm C18 resin. Separation was performed at a constant flow rate of 300 nl/min using mobile phases consisting of 0.1% FA in water (Buffer A) and 0.1% FA in 80% acetonitrile (Buffer B). A 40-min optimized gradient was employed: 5% B initially for 2 min, increasing linearly to 35% B over 26 min, followed by a ramp to 90% B within 4 min. The column was then washed with 90% B for 8 min. The column was then re-equilibrated at 5% B before the next injection.

The UHPLC was coupled via a Nanospray Flex Ion Source (spray voltage: 2150 V; ion transfer tube: 320 °C) to an Orbitrap Eclipse Tribrid mass spectrometer (Thermo Fisher Scientific). Data acquisition utilized a data-dependent acquisition (DDA) strategy with a 2.5 s cycle time. Full MS scans were acquired in the Orbitrap at a resolution of 60,000 (at m/z 200) over an m/z range of 400 to 1800. The automatic gain control (AGC) target was set to standard, and monoisotopic peak determination was enabled for peptides. Precursors with charge states 2 to 7 were isolated with a 0.8 m/z window and fragmented using both higher-energy collisional dissociation (HCD) (NCE 30%) and electron-transfer/higher-energy collision dissociation (EThcD). EThcD spectra were acquired at supplemental activation collision energies of 25%, 55%, and 75%. MS/MS spectra were acquired in the Orbitrap at a resolution of 15,000 with an AGC target of 5 × 10^4^. Dynamic exclusion was enabled for 10 s after one observation with a mass tolerance of ±8 ppm. Leucine (Leu) and isoleucine (Ile) assignments were inferred from the cleavage preferences of pepsin and chymotrypsin, as described previously (23).

*De novo* sequencing and *de novo*-assisted database searching (PEAKS DB) were performed using the *De Novo* and PEAKS DB workflows in PEAKS Studio 12.5 (build 20241120; Bioinformatics Solutions Inc). Search parameters included carbamidomethylation (C, +57.02 Da) as a fixed modification, and oxidation (M, +15.99 Da), deamidation (N, +0.98 Da), and N-terminal pyroglutamate formation (Q, −17.03 Da) as variable modifications, with a maximum of 2 variable PTMs allowed per peptide. The mass tolerances for precursor and fragment ions were set to 10 ppm and 0.02 Da, respectively. For database searching, each dataset was searched using its corresponding enzyme specificity in semispecific mode, allowing up to two missed cleavages. For the *Nepenthes* dataset, the enzyme was set to “None” and the digest mode to “Unspecific”. The peptide length was restricted to 6 to 45 amino acids. To maximize identification sensitivity and coverage, SPIDER algorithm (for mutation and sequence variant identification), and PEAKS PTM (for unspecified modification blind searching) were enabled. Database-search results were filtered at a 1.0% false discovery rate (FDR) at both the peptide-spectrum match (PSM) and protein levels. All *de novo* peptide sequences with an average local confidence score ≥80% in PEAKS Studio were manually validated by inspecting the corresponding MS/MS spectra for adequate fragment-ion support (Supplemental Table S6).

The overlapping peptides were assembled to generate full-length light-chain sequences. Finally, the assembled sequences were appended to *Homo sapiens* reference proteome from UniProt (https://www.uniprot.org/) and the Amyloid Light Chain Database (https://wwwapp.bumc.bu.edu/BEDAC_ALBase/Search) (24), and the corresponding MS/MS datasets were analyzed using Novor.cloud (Rapid Novor Inc., https://novor.cloud/) to confirm peptide coverage across the sequences as previously described (Supplemental Table S7) (17, 25).

### RNA-Based Immunoglobulin Repertoire Sequencing

Following our established protocol, total RNA was extracted from bone marrow mononuclear cells using TRIzol, and immunoglobulin repertoire sequencing was performed by SeqHealth (26). Libraries were prepared using the KC-Digital Stranded BCR-seq Library Prep Kit for Illumina according to a 5′ rapid amplification of cDNA ends (5′ RACE) protocol incorporating unique molecular identifiers (UMIs). The resulting libraries were sequenced on a NovaSeq X Plus system (Illumina). Full-length light-chain variable-region sequences spanning the V–J rearrangement were assembled using MiXCR and annotated using IMGT/HighV-QUEST (https://www.imgt.org/). Detailed criteria for clonotype definition, clonotype-frequency calculation, and dominant-clonotype identification are provided in the Supplementary Methods of our previous publication (26).

### Histological Laser Microdissection of Renal Amyloid Deposits and MS Analysis

Renal biopsy tissues were fixed in 10% neutral buffered formalin, paraffin-embedded, and sectioned at 6 μm onto PEN membrane slides. The sections were stained with Congo red for 17 min and differentiated in alkaline ethanol for 3 s. Congo red–positive amyloid deposits in glomeruli, interstitial regions, and blood vessels were microdissected using a Leica LMD6000 system, with each sample providing at least 600,000 μm^2^ of tissue. Microdissected material was collected into 0.5-ml tubes containing 20 μl Tris/EDTA/0.002% Zwittergent. The microdissected material was reduced with DTT, alkylated with IAA, and deglycosylated with PNGase F. The samples were then divided into two aliquots and digested separately with trypsin or chymotrypsin.

Peptide samples were analyzed with Orbitrap Eclipse Tribrid mass spectrometer (Thermo Fisher Scientific). Peptides were separated on an in-house packed reversed-phase capillary column. Solvent A consisted of water containing 0.1% FA, and solvent B consisted of acetonitrile containing 0.1% FA. The liquid chromatography gradient was as follows: 2% solvent B for 2 min, increased to 10% B over 1 min, ramped to 25% B over 35 min, then increased to 40% B over 25 min. The column was subsequently washed with 99% B for 10 min and re-equilibrated at 2% B for 5 min.

The Orbitrap Eclipse mass spectrometer was operated in DDA mode with a 3 s cycle time. Full MS scans were acquired in the Orbitrap over an m/z range of 350 to 1600 at a resolution of 60,000, with an AGC target of 4 × 10^5^ and the maximum injection time set to Auto. The most abundant precursor ions with charge states of 2 to 7 were isolated using a 1.6 m/z isolation window and fragmented by HCD with a normalized collision energy of 30%. MS/MS spectra were acquired in the Orbitrap at a resolution of 15,000, with an AGC target of 5 × 10^4^ and injection time set to Auto. Dynamic exclusion was enabled with an exclusion duration of 15 s, with a mass tolerance of ±10 ppm. Isotope exclusion was enabled. Only precursor ions with intensities above 5 × 10^4^ were selected for fragmentation.

LC–MS/MS data were searched against a customized database comprising our group's in-house light-chain protein sequence database (including the eight urine-assembled light-chain sequences generated in this study), the *H. sapiens* reference proteome from UniProt (https://www.uniprot.org/) and the Amyloid Light Chain Database (https://wwwapp.bumc.bu.edu/BEDAC_ALBase/Search) (both downloaded in January 2025) (24). Database searching was performed in parallel using the novor.cloud platform and the PEAKS DB workflow implemented in PEAKS Studio 12.5 (Bioinformatics Solutions Inc) (25) (Supplemental Tables S8–S11). Enzyme specificity was set according to the corresponding protease (trypsin or chymotrypsin) for each dataset. Carbamidomethylation (C) was applied for fixed modifications, whereas variable modifications included oxidation (M), deamidation (N), and N-terminal pyroglutamate formation (Q). Precursor and fragment ion mass tolerances were restricted to 10 ppm and 0.02 Da, respectively. Finally, database-search results were filtered to maintain a 1% FDR. The resulting peptide identifications were used to assess the identification of the urine-derived assembled light-chain sequences and their V_L_ coverage in renal amyloid deposits. Representative annotated MS/MS spectra were visualized and exported using the Interactive Peptide Spectral Annotator (IPSA) (http://interactivepeptidespectralannotator.com) for figure preparation (27).

### Structural Modeling and Pairwise Alignment

Following our previously described method (28), AlphaFold 3 was used to independently generate N-acetylglucosamine (NAG)-containing and glycan-free models from the same amino acid sequence for each light chain. In the NAG-containing model, NAG, the innermost sugar directly linked to Asn, was specified as an Asn-linked PTM at the experimentally identified glycosylation site and modeled jointly with the protein to represent the local structural context of N-glycosylation. Corresponding NAG-containing and glycan-free models were aligned using TM-align implemented in the RCSB PDB Pairwise Structure Alignment Tool. Root-mean-square deviation (RMSD) was calculated between aligned Cα atom pairs after structural superposition, with lower values indicating greater structural similarity. The template modeling score (TM-score) ranges from 0 to 1 and assesses global structural similarity, with higher values indicating greater similarity in the overall fold.

## Results

### Analytical Workflow for Amyloidogenic Light-Chain Sequencing and Tissue Validation

Building on previous mass spectrometry–based approaches for light-chain sequencing, we implemented an analytical workflow for AL amyloidosis (Fig. 1). Intact mass measurements before and after deglycosylation were used to determine N-glycosylation status and obtain the post-deglycosylation masses of the light chains. The resulting deglycosylated intact masses were then used to constrain candidate sequence selection. Following deglycosylation, parallel digestions using a panel of proteases generated overlapping peptides spanning both the V_L_ and C_L_ domains, enabling *de novo* sequencing and full-length light chain sequence assembly. The assembled sequences were evaluated by comparing the experimental intact masses with the theoretical molecular masses calculated from the assembled sequences. For peptide-level confirmation, the sequences were appended to a customized database, and the corresponding MS/MS datasets were searched against this database. Finally, proteomic analysis of laser-microdissected renal amyloid deposits identified the patient-matched urine-derived sequence as the highest-scoring light-chain sequence within the clinically determined amyloidogenic isotype (κ or λ), supporting its incorporation into amyloid fibrils.

**Fig. 1:**
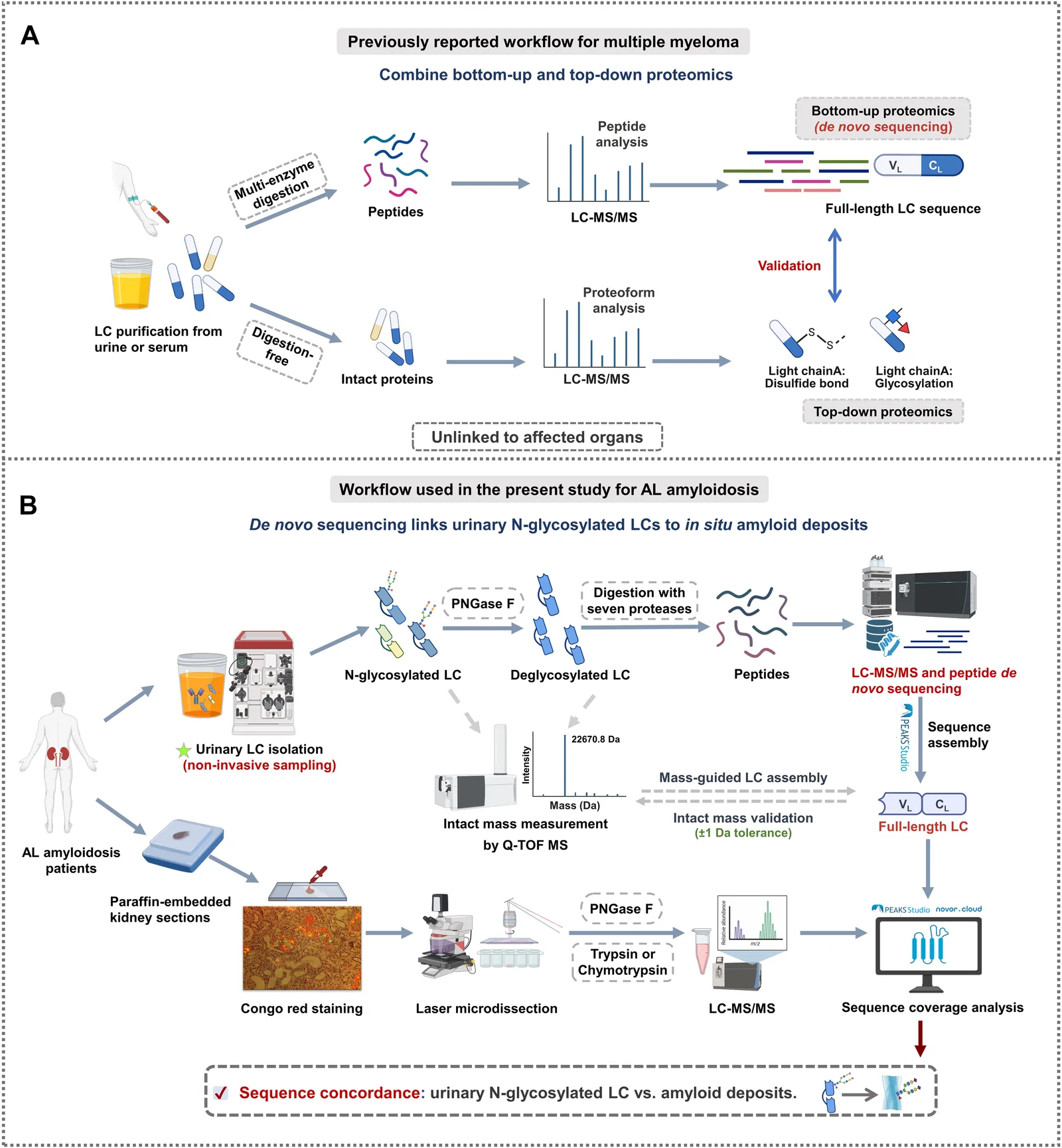
**Comparison of a previously reported light-chain sequencing workflow for multiple myeloma and the workflow used in the present study for AL amyloidosis.***A*, previously reported workflow for multiple myeloma (16), in which abundant monoclonal light chains isolated from urine or serum were analyzed by complementary bottom-up multi-enzyme de novo sequencing and top-down proteoform analysis. This workflow is shown for comparison with the workflow used in the present study. *B*, integrated workflow employing *de novo* peptide sequencing for urinary light chains with proteomic analysis of *in situ* amyloid deposits. Purified urinary light chains were analyzed by Q-TOF LC–MS to determine deconvoluted molecular masses before and after PNGase F treatment. The light chains were then digested separately with seven proteases and analyzed by LC-MS/MS for *de novo* peptide sequencing. Full-length light-chain sequences were assembled using the measured intact mass as a constraint and validated by database searching. Concurrently, amyloid deposits are collected from kidney sections via laser microdissection. The assembled light-chain sequence is then integrated into a customized protein database to verify peptide coverage within the amyloid deposits, providing evidence that N-glycosylated light chains are incorporated into amyloid fibrils.

### Confirmation of N-glycosylation by Intact Mass Measurement Following PNGase F Treatment

The theoretical masses for unglycosylated λ and κ light chains typically range from 22,200 to 23,100 Da and 23,100 to 24,600 Da, respectively (29). In this study, the measured intact masses of glycosylated light chains consistently exceeded these ranges (λ > 24,000 Da; κ > 25,000 Da). Following enzymatic deglycosylation, the intact masses decreased by approximately 1600 to 2300 Da (Fig. 2) (Supplemental Table S2), consistent with a single occupied N-glycosylation site in each light chain (30). Comparison of the observed mass differences with entries in the GlycoStore database suggested the presence of biantennary complex-type N-glycans (31). Before deglycosylation, intact light chains frequently exhibited multiple glycoforms. In a subset of samples, adjacent glycoforms differed in molecular mass by approximately 203 and 291 Da, consistent with compositional differences of one HexNAc or Neu5Ac residue, respectively. However, these intact mass measurements can infer only glycan composition, not the actual glycan structure.

**Fig. 2:**
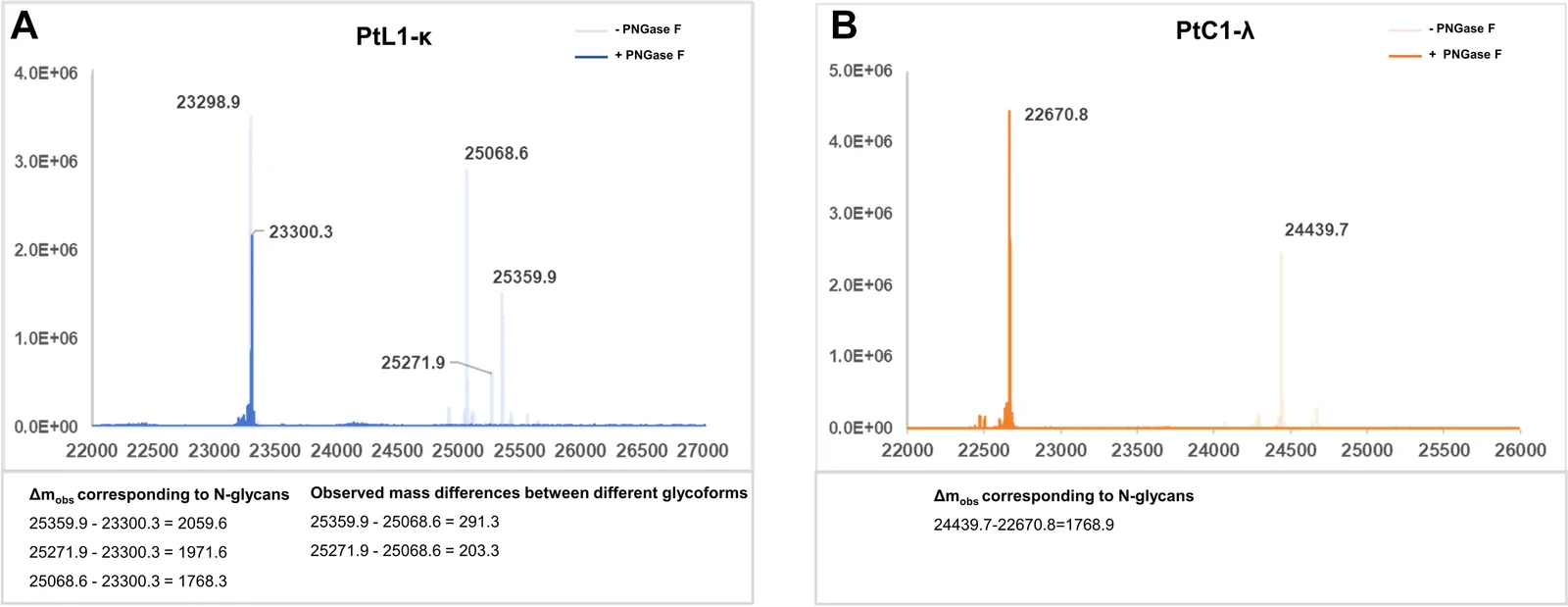
**Deglycosylation of monoclonal light chains with PNGase F.***A* and *B*, intact mass spectra of PtL1-κ and PtC1-λ under reducing conditions. PtL1-κ exhibits an unglycosylated peak at 23,298.9 Da alongside multiple glycosylated light chains; PtC1-λ shows a dominant glycosylated species at 24,439.7 Da. PNGase F treatment resolves both light chains to single major peaks (23,300.3 Da and 22,670.8 Da, respectively), confirming the removal of N-glycans. The +1.4 Da shift in the PtL1-κ (23,300.3 vs. 23,298.9) is approximately consistent with Asn deamidation upon enzymatic deglycosylation.

### Full-Length Sequence Assembly and N-Glycosylation Site Determination of the Amyloidogenic Light Chains

To determine the full-length sequences of amyloidogenic light chains, we applied a bottom-up workflow combining multi-enzyme digestion, high-confidence *de novo* peptide sequencing, and database searching (Fig. 3*A*). Comparison of the PtC1-λ datasets showed that peptides spanning the glycosylated region were poorly detected without PNGase F treatment, leaving persistent sequence-coverage gaps despite parallel multi-enzyme digestion (Supplemental Fig. S1, *A* and *C*). Following PNGase F treatment, peptides spanning the glycosylated region were detected in the deamidated form at the candidate N-glycosylation site, facilitating both sequence assembly and site localization (Supplementary Fig. S1, *B* and *D*). Based on these findings, a pre-digestion deglycosylation step was standardized for all subsequent samples to improve the sequence coverage. Peptides identified from multi-enzyme LC-MS/MS data were mapped onto immunoglobulin germline segments. In the representative case, the V_L_ sequence was assigned to the immunoglobulin kappa variable 1 to 39 (*IGKV1-39*) and immunoglobulin kappa joining 1 (*IGKJ1*) germline gene segments, whereas the C_L_ sequence matched the 01 allele of the immunoglobulin kappa constant gene (*IGKC∗01*). Candidate full-length sequences were then iteratively refined using post-deglycosylation intact-mass constraints and confirmed by database reanalysis (Fig. 3*A*).

**Fig. 3:**
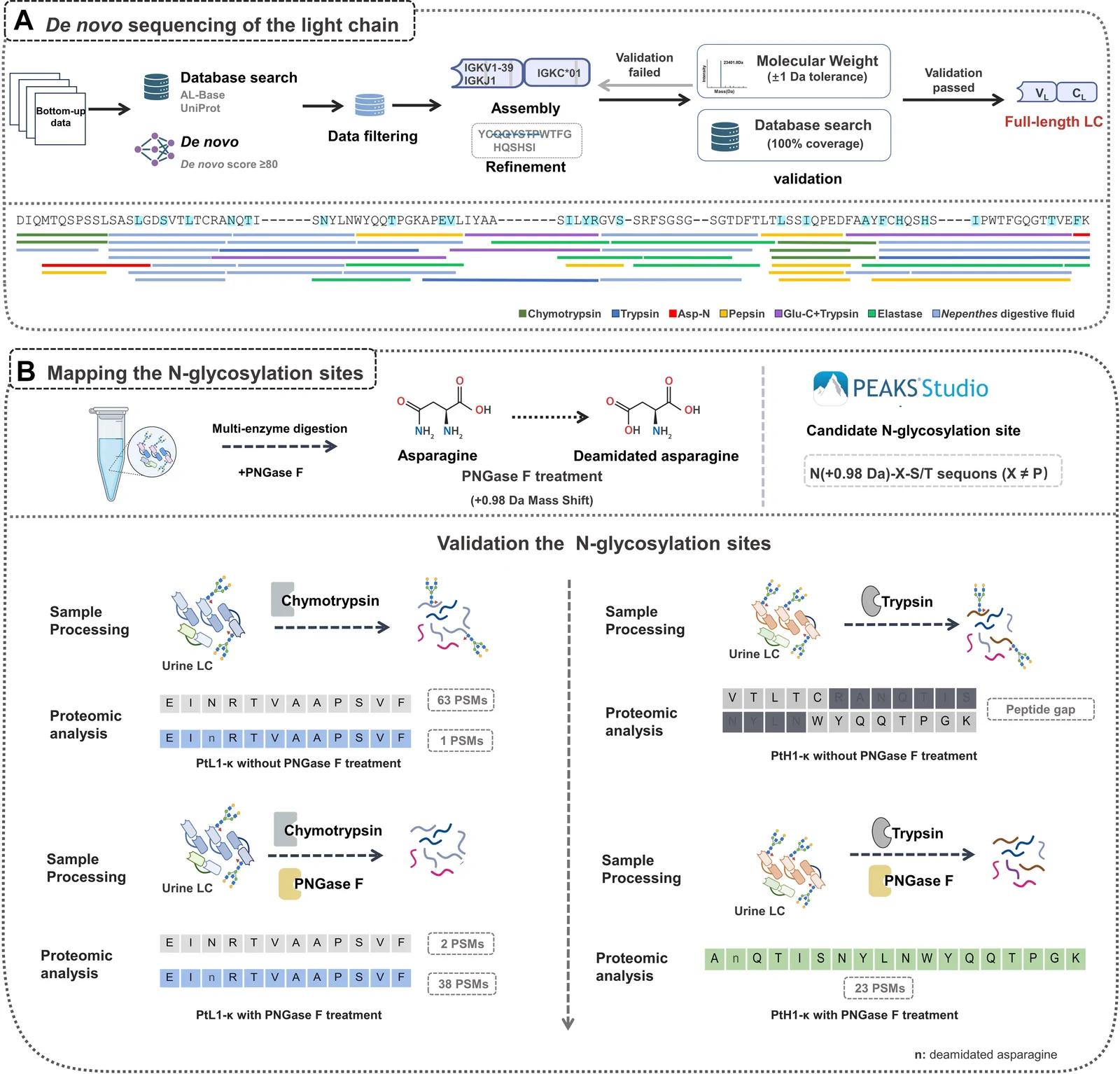
**Strategy for the comprehensive *de novo* sequencing of amyloidogenic light chains and mapping of N-glycosylation sites.***A*, Bottom-up proteomic workflow for full-length light chain sequence reconstruction. Raw MS data undergo concurrent database searching and *de novo* sequencing. Following data filtering, sequence assembly and refinement, candidate sequences are validated using intact mass and database search validation to ensure complete sequence representation. The *lower panel* provides a schematic representation of the sequence assembly strategy (PtH1-κ), demonstrating how overlapping peptides generated via a multi-enzyme digestion approach (including Chymotrypsin, Trypsin, Asp-N, Pepsin, Glu-C + trypsin, Elastase, and Nepenthes digestive fluid) are assembled to achieve full-length light chain coverage. Amino acid substitutions relative to the germline sequence are highlighted in *blue*. *B*, workflow for systematic mapping and validation of N-glycosylation sites. Potential N-glycosylation sites were identified in PEAKS Studio by requiring PNGase F–induced deamidated Asn within the canonical N-X-S/T sequon (X ≠ P). Site assignments were further evaluated by comparing peptides identified before and after PNGase F treatment following digestion with the same protease. PNGase F treatment either increased the detection of deamidated peptides, as reflected by higher PSM counts, or enabled detection of a previously undetected peptide region, supporting assignment of the corresponding N-glycosylation sites.

After sequence assembly, N-glycosylation sites were localized using a PNGase F–based comparative strategy (Fig. 3*B*). Candidate sites were assigned based on two stringent criteria: localization within the canonical N-X-S/T sequon (X ≠ P) and detection of the characteristic +0.98 Da mass shift corresponding to PNGase F–induced Asn deamidation. The assignment of each site was supported by the emergence of deamidated peptides that precisely resolved previous coverage gaps in regions where signals were either undetected or detected predominantly with the sequon Asn unmodified in the corresponding untreated samples (Supplemental Figs. S3–S9, Supplemental Table S12). For example, in PtL1-κ, the detected peptides shifted from predominantly unmodified to deamidated forms after PNGase F treatment, whereas in PtH1-κ, treatment enabled detection of a deamidated peptide spanning a region that was not detected in the untreated sample (Fig. 3*B*). Together, these paired MS/MS results supported the localization of N-glycosylation sites in the amyloidogenic light chains.

### Comprehensive Validation of Assembled Light Chain Sequences

To validate the assembled light chain sequences, we implemented two complementary mass spectrometry–based approaches: intact-mass validation and peptide-level sequence confirmation (Fig. 3*A*). For intact-mass validation, theoretical molecular masses were calculated from the assembled sequences with relevant sequence-specific modifications taken into account, as described in Method section. In all cases, the theoretical molecular masses agreed with the experimentally measured masses within ±1 Da (Supplemental Table S2), confirming sequence completeness and assembly accuracy (16, 32). For peptide-level validation, the assembled sequences were incorporated into a customized database for searching the acquired bottom-up MS/MS datasets of urine-derived light chains. In every case, the reconstructed sequence was identified as the highest-scoring match with 100% sequence coverage (Supplemental Fig. S10).

To provide orthogonal transcript-level validation, RNA-based immunoglobulin sequencing was performed in two patients for whom stored bone marrow samples were available for sequencing, including one with AL-λ (PtC1-λ) and one with AL-κ (PtZ2-κ). The MS-derived amino acid sequences spanning the V_L_ and partial C_L_ regions showed 100% identity with the corresponding transcript-derived sequences (Supplemental Table S3).

### Amino Acid Substitutions and N-Glycosylation Sites in Amyloidogenic Light Chains

We determined the full-length amino acid sequences of the amyloidogenic light chains from eight patients (Supplemental Fig. S2). Each assembled sequence was compared with reference sequences representing the inherited forms of human immunoglobulin genes in IMGT, the international ImMunoGeneTics information system, which provides standardized references and numbering for immunoglobulin sequence analysis. This comparison enabled assignment of the closest-matching V, J, and C gene segments. As summarized in Figure 4*A*, among the κ cases, one was assigned to *IGKV1-16* (PtL1-κ), two to *IGKV1-33* (PtZ1-κ and PtZ2-κ), two to *IGKV1-39* (PtH1-κ and PtB1-κ), and one to *IGKV4-1* (PtB2-κ). Both λ light chains were assigned to *IGLV2-14* (PtC1-λ and PtT1-λ).

**Fig. 4:**
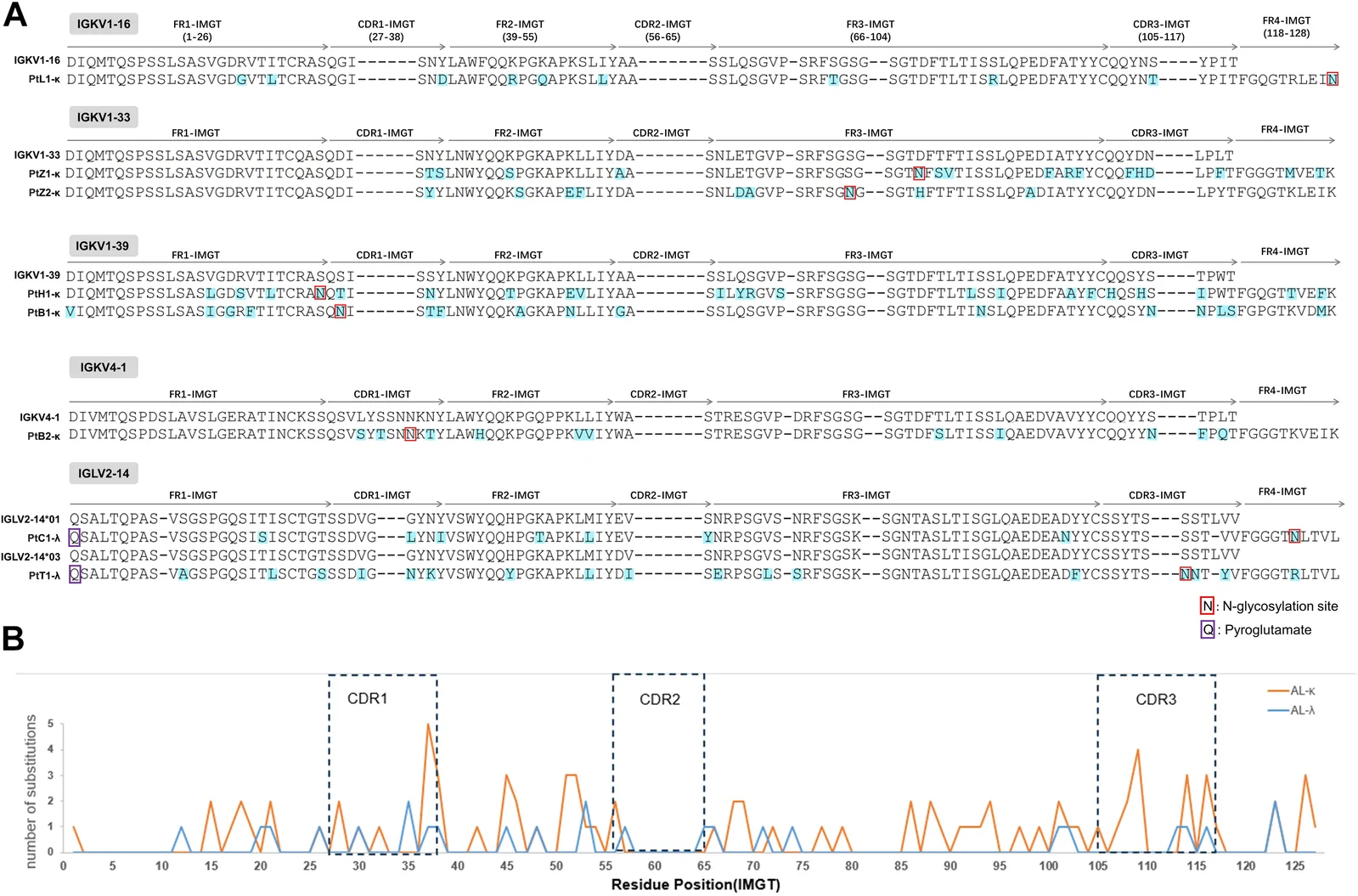
**Sequence alignment and amino acid substitution profiling of amyloidogenic light chain V_L_ domains.***A*, reconstructed amino acid sequences of patient-derived amyloidogenic light-chain V_L_ domains were aligned with their closest-matching IMGT germline V- and J-gene reference sequences and grouped according to V-gene assignment. Amino acid substitutions relative to the assigned germline references are highlighted in *blue*, N-glycosylation sites in *red boxes*, and N-terminal pyroglutamate residues in *violet boxes*. *B*, positional distribution of amino acid substitutions across the light-chain V_L_ domain. The line plot shows the number of sequences containing a substitution at each IMGT amino acid position. AL-κ and AL-λ sequences are shown in *orange* and *blue*, respectively. *Dashed boxes* indicate the CDR1, CDR2, and CDR3 regions. Only mismatches at aligned positions were counted, whereas insertions and deletions at the V–J junction were excluded.

Using these assigned germline references, we next assessed amino acid substitutions within the V_L_ domain. Standardized IMGT numbering assigns equivalent positions across different light-chain sequences, allowing substitution sites to be compared among patients. The mutation burden ranged from 8 to 22 amino acid substitutions per sequence (Fig. 4*B*). Of all substitutions in the κ sequences, 28.6% were localized in FR3, followed by FR2 and CDR3 (17.9% each), and CDR1 (14.3%). Notably, IMGT position 37 within CDR1 was the most recurrently substituted position, exhibiting five independent substitutions. In the two λ light chains, IMGT positions 35 (CDR1), 53 (FR2), and 123 (FR4) were substituted in both sequences.

In addition to these amino acid substitutions, N-glycosylation sites were also identified at multiple positions across the V_L_ domain. These sites were distributed across several functional regions, including FR1 (PtH1-κ), FR3 (PtZ1-κ, PtZ2-κ), FR4 (PtL1-κ, PtC1-λ), CDR1 (PtB1-κ, PtB2-κ), and CDR3 (PtT1-λ) (Table 1).

**Table 1:** N-glycosylation sequons identified in patient-derived light chains

Case	Region	Pre-mutation sequence	N-glycosylation sequon
PtL1-κ	FR4	KRT	NRT
PtZ1-κ	FR3	DFT	NFS
PtZ2-κ	FR3	SGS	NGS
PtH1-κ	FR1	SQS	NQT
PtB1-κ	CDR1	SIS	NIS
PtB2-κ	CDR1	NKN	NKT
PtC1-λ	FR4	KLT	NLT
PtT1-λ	CDR3	SST	NNT

### Sequence Concordance of Urine-Derived Light Chains with Renal Amyloid Deposits

To verify the concordance between urine-assembled light chain sequences and renal amyloid deposits, Congo red-positive amyloid deposits from paraffin-embedded renal biopsy sections were collected by laser microdissection and analyzed by LC–MS/MS after parallel digestion with trypsin and chymotrypsin (Fig. 5, *A* and *B*). Tissue proteomic datasets were analyzed using both novor.cloud and PEAKS. For seven patients, the corresponding urine-assembled sequence ranked first among all light-chain identifications in each workflow for both the tryptic and chymotryptic datasets (Supplemental Figs. S11 and S12, Supplemental Tables S4 and S5). For PtC1-λ, both workflows identified the corresponding sequence as the highest-scoring λ-chain identification in the tryptic dataset, whereas only novor.cloud identified the corresponding sequence in the chymotryptic dataset (Supplemental Table S5). Across the cohort, combined analysis of the tryptic and chymotryptic datasets yielded 87.6% to 100% coverage of the urine-assembled light chain V_L_. This coverage was defined as the proportion of residues in the urine-assembled V_L_ sequence covered by peptides identified in the renal amyloid deposits (Fig. 6*A*, Supplemental Fig. S13). This extensive coverage encompassed multiple mutated residues unique to the patient's specific light chain, demonstrating strong concordance between the urinary light-chain sequence and the light chain in renal amyloid deposits.

**Fig. 5:**
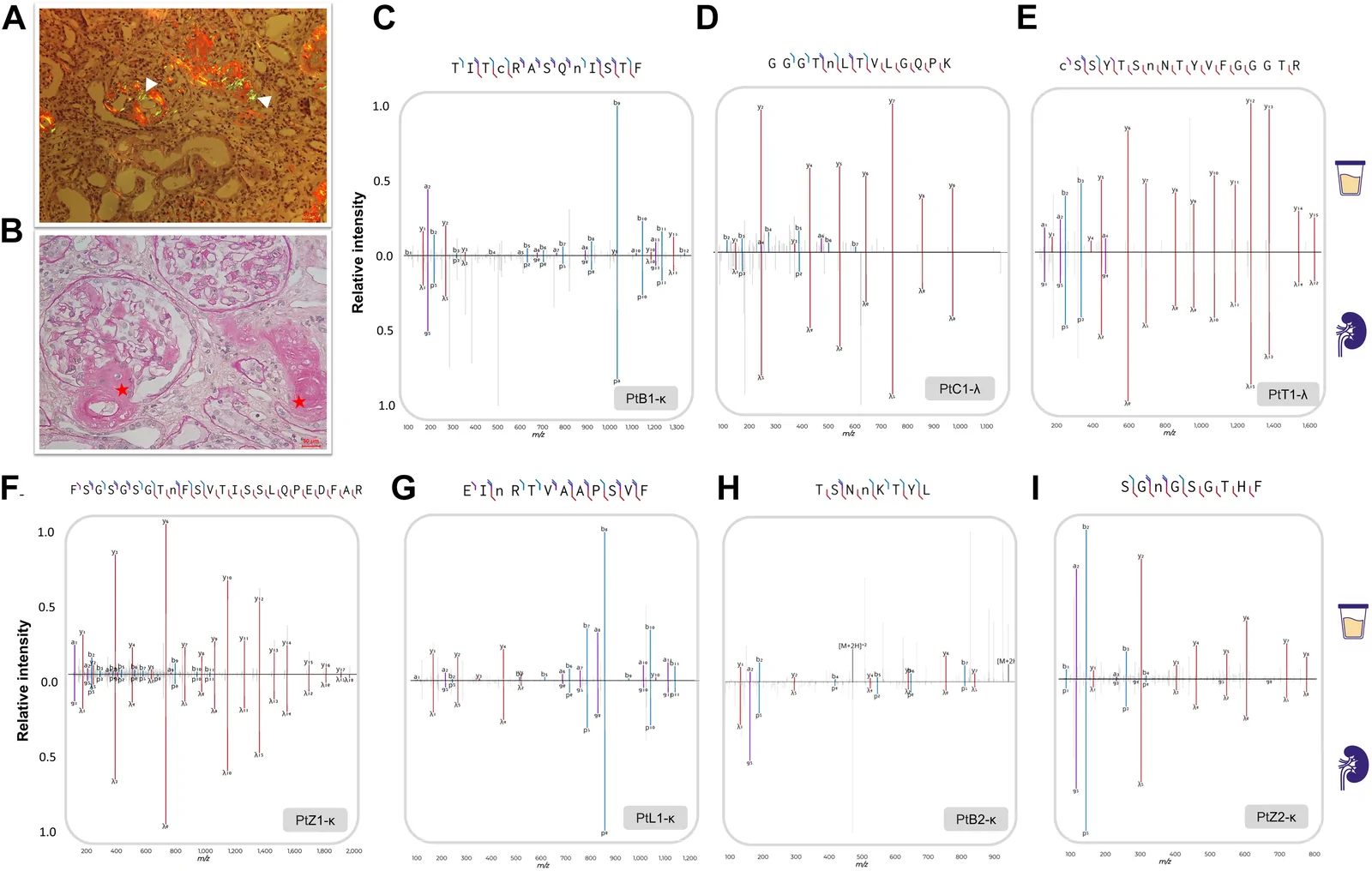
**Histological demonstration of renal amyloid deposits and detection of deamidated peptides at previously identified urinary light-chain N-glycosylation sites.***A*, representative renal biopsy section stained with Congo red and viewed under polarized light, showing characteristic birefringence of the amyloid deposits (*white arrowhead*). *B*, periodic acid–Schiff (PAS) staining of the same biopsy specimen, showing amyloid deposits (*red stars*) with staining intensity comparable to that of Bowman's capsule. *C–I*, mirrored MS/MS spectra of peptides spanning N-X-S/T sequons (X ≠ P) identified in PNGase F-treated urinary light chains (*top*) and renal amyloid deposits (*bottom*). The corresponding sites had previously been confirmed as N-glycosylated in the urinary light chains. Following PNGase F treatment, peptides containing deamidated Asn, denoted by “n,” were identified at the same positions in the renal amyloid deposits. The highly similar fragmentation patterns support assignment of the same peptide sequences in urine and tissue. The corresponding spectra for PtH1-κ are shown in Fig. 5*B*.

**Fig. 6:**
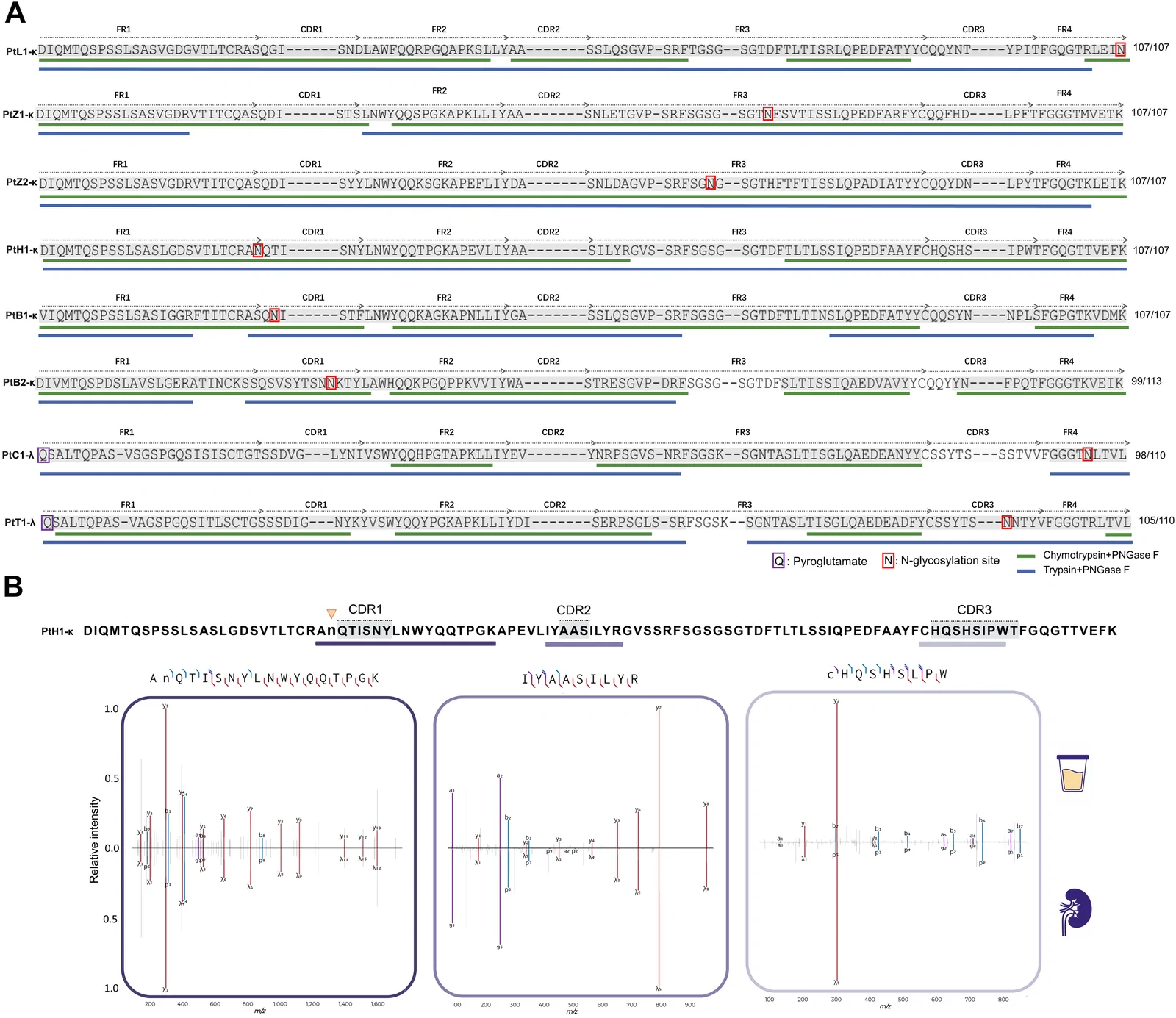
**Concordance of urine-derived light chain with renal amyloid deposits.***A*, V_L_-domain coverage of the assembled light-chain sequences in renal amyloid deposits. Amino acid sequences detected by microdissected renal deposit proteomics are highlighted in *gray*. *Blue* and *green bars* represent peptides identified via trypsin and chymotrypsin digestion, respectively. N-glycosylation sites are highlighted in *red boxes*. Pyroglutamate residues are highlighted in *viole*t *boxes*. The PtC1-λ chymotrypsin dataset was the only exception. This dataset was derived from a renal biopsy specimen with minimal amyloid deposition; novor.cloud identified the corresponding urine-assembled light-chain sequence, whereas PEAKS DB did not. Therefore, for consistency, peptide coverage for all samples was displayed based on novor.cloud analysis. The corresponding V_L_-coverage maps exported directly from PEAKS Studio have also been added as Supplemental Figure S13. *B*, mirrored peptide fragmentation spectra covering the CDR1, CDR2, and CDR3 regions, comparing urine-assembled light chain (*top*) with corresponding peptides detected in renal amyloid deposits (*bottom*). N-glycosylation site is specifically indicated by an *orange inverted triangle*. Spectra are annotated with a-ions (*purple*), b-ions (*blue*), and y-ions (*red*).

To provide an intuitive demonstration of this consistency, we focused on sample PtH1-κ, which exhibited the highest substitution burden relative to the germline sequence. Mirror plots were generated to compare representative MS/MS spectra of peptides spanning the highly variable CDR1, CDR2, and CDR3 regions between peptides from the urine-assembled light chain and the corresponding peptides in renal amyloid deposits. The peptide spanning the FR1–CDR1 also contained the N-glycosylation site. These 3 MS/MS comparisons showed similarities both in the m/z positions of the b- and y-ion series and their relative fragment ion intensities (Fig. 6*B*). Across all eight cases, N-glycosylation had been experimentally confirmed at each site in the matched urinary light chains. Following PNGase F treatment of laser-microdissected renal amyloid deposits, peptides spanning the corresponding sites were consistently identified with deamidated Asn (Figs. 5, *C–I* and 6*B*). Taken together, these results support the involvement of N-glycosylated light chains in amyloid fibril formation.

### Structural Mapping of *De Novo* Sequenced Light Chains and Their N-Glycosylation Sites

Having determined the full-length light-chain sequences and N-glycosylation sites, we used AlphaFold 3 to model the structures of PtB1-κ and PtZ2-κ. Their glycosylation sites were located in CDR1 and FR3 regions, respectively; these regions have been reported as recurrent locations for acquired N-glycosylation sites in AL amyloidosis and MM (33). In PtB1-κ, a Ser-to-Asn substitution generated an Asn-Ile-Ser (NIS) N-glycosylation sequon at residue Asn28 (IMGT position 28) within the flexible CDR1 loop. In PtZ2-κ, a Ser-to-Asn substitution generated an Asn-Gly-Ser (NGS) N-glycosylation sequon at Asn65 (IMGT position 79) within an FR3 β-strand. In the PtZ2-κ model, the innermost NAG linked to Asn65 formed a putative 3.0 Å polar contact with Arg18 (R18), suggesting a possible local glycan–protein interaction (Fig. 7, *A* and *B*).

**Fig. 7:**
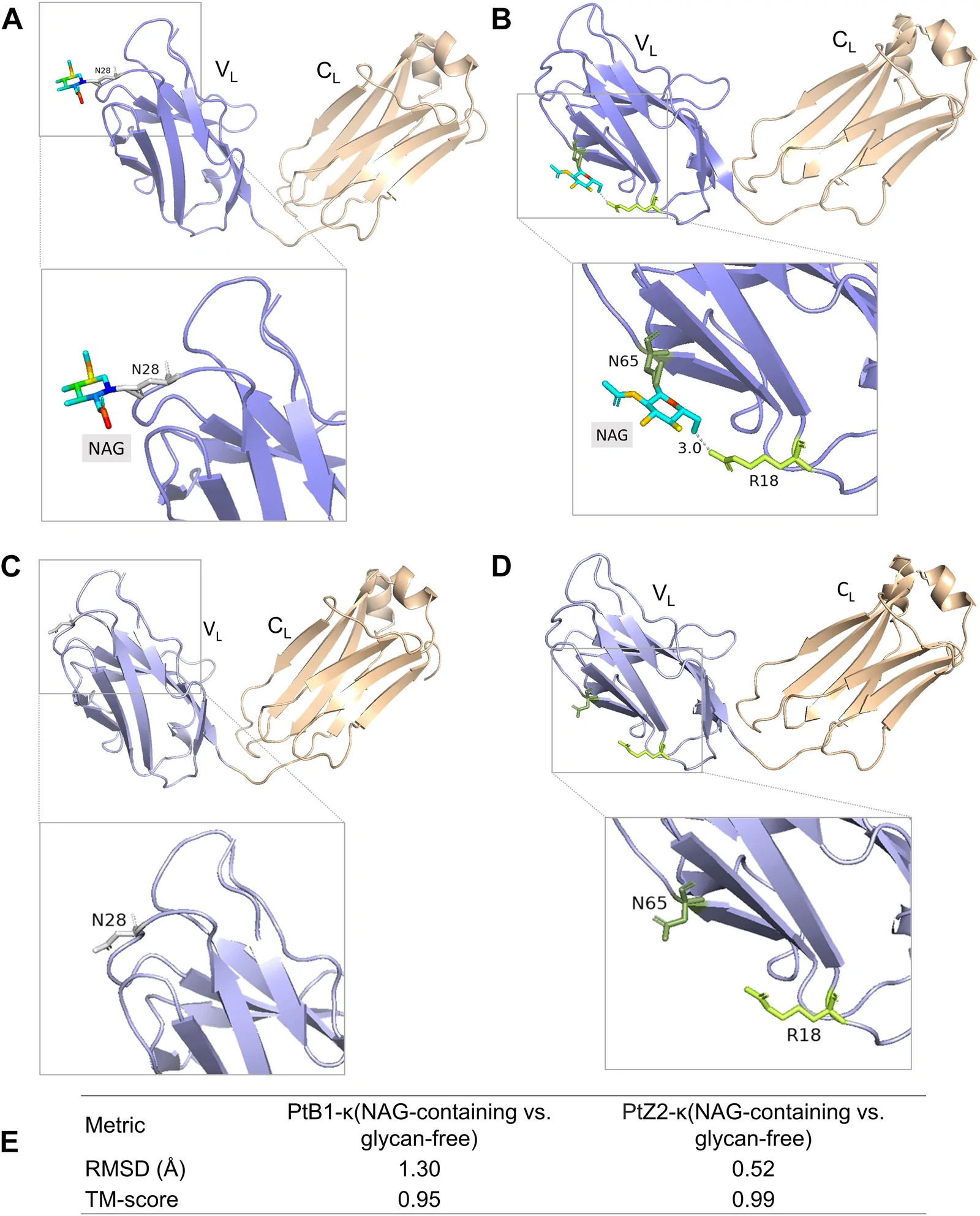
**Structural comparison of NAG-containing and glycan-free amyloidogenic light-chain models.***A* and *B*, predicted full-length structures of PtB1-κ and PtZ2-κ containing the innermost NAG residue directly linked to the glycosylated Asn. Only the innermost NAG was modeled because the complete site-specific glycan structures were not experimentally resolved. The V_L_ is shown in *blue* and the C_L_ in *gold*. NAG is shown as *sticks*, with carbon atoms colored *cyan* and heteroatoms colored according to element. Enlarged views show (*A*) the CDR1 N-glycosylation site Asn28 (N28) in PtB1-κ, shown as light-grey sticks, and (*B*) the FR3 N-glycosylation site Asn65 (N65) in PtZ2-κ, shown as *dark-green sticks*. In the PtZ2-κ model, NAG linked to Asn65 forms a putative 3.0 Å polar contact with Arg18 (R18); this contact is indicated by a *dashed line*. *C* and *D*, corresponding glycan-free models of (*C*) PtB1-κ and (*D*) PtZ2-κ. *E*, the PtB1-κ models showed an RMSD of 1.30 Å and a TM-score of 0.95, whereas the PtZ2-κ models showed an RMSD of 0.52 Å and a TM-score of 0.99. Both pairs therefore retained highly similar global folds, although the aligned Cα atoms showed greater positional deviation in PtB1-κ than in PtZ2-κ. Residue labels N28, N65, and R18 refer to positions in the light-chain amino acid sequences.

Comparison of each NAG-containing model with its glycan-free counterpart showed similar overall folds. The greater structural difference between the PtB1-κ models than between the PtZ2-κ models (RMSD, 1.30 vs 0.52 Å) may be related to the location of the PtB1-κ glycosylation site within the conformationally flexible CDR1 loop (Fig. 7, *C–E*). Together with the putative NAG–R18 contact in PtZ2-κ, these model-specific differences suggest that N-glycosylation may exert local structural effects that vary with the light-chain sequence and the structural context of the glycosylation site. Consistent with this possibility, a recent hydrogen–deuterium exchange mass spectrometry study showed that the effects of N-glycosylation on deuterium uptake varied across light-chain regions and among individual light chains (34). Further experimental studies are required to determine the extent, underlying mechanisms, and functional significance of these structural effects.

## Discussion

In this study, we implemented an integrated analytical workflow for urinary light-chain analysis using *de novo* peptide sequencing tools. This workflow enables the characterization of patient-specific, light-chain sequences and N-glycosylation sites at amino acid resolution from routine clinical specimens. Furthermore, proteomic analysis of renal amyloid deposits supported the corresponding urinary light chains as precursor proteins for renal amyloid formation. Together, this workflow provides a practical approach for linking circulating monoclonal immunoglobulin light chains with tissue amyloid deposits and may facilitate mechanistic studies across the broader spectrum of light chain-related diseases.

*De novo* sequencing has progressed from the sequence verification of purified recombinant antibodies to resolving full-length, individual monoclonal IgG1 clones directly from the complex polyclonal background of human serum (35, 36). Consequently, this technology has become indispensable for characterizing pathogenic immunoglobulins in plasma cell dyscrasias. Dupré *et al.* and Wang *et al.* reconstructed full-length monoclonal light chains directly from patient urine in MM and cast nephropathy (16, 17). Peng *et al.* mapped complete antibody sequences to localize critical modifications, such as Fab glycosylation within the heavy-chain CDR1 (37). Furthermore, in MM, *de novo* sequencing can identify patient-specific clonotypic peptides in hypervariable CDR regions at diagnosis, enabling their subsequent targeted tracking (38, 39). To date, *de novo* sequencing efforts in AL amyloidosis have been predominantly restricted to tissue-extracted amyloid fibrils, primarily of the λ isotype (40, 41). Because these analyses are performed on deposited fibrils, recovery of full-length precursor sequences is generally not feasible, limiting recombinant expression and downstream functional studies (42). Information on N-glycosylated light chains, which are more prevalent in κ-type AL amyloidosis, also remains limited. These limitations underscore the importance of directly characterizing patient-specific circulating free light chains in AL amyloidosis, including their full-length sequences and PTMs.

The typically low clonal plasma cell burden in AL amyloidosis results in limited purity of circulating monoclonal free light chains, complicating *de novo* sequence reconstruction. More importantly, the most abundant urinary monoclonal light-chain species cannot be assumed to be the amyloidogenic precursor solely based on its predominance in circulation (14). Definitive identification therefore requires matching the urinary light-chain sequence to that detected in tissue amyloid deposits. Proteomic analysis of laser-microdissected deposits remains the gold standard for this verification (14, 43). Without the patient-specific light-chain sequence in the search database, proteomic analysis of renal amyloid deposits identified mainly conserved light-chain peptides. Incorporation of the urine-assembled full-length sequence enabled extensive mapping of the variable region, including most patient-specific mutations, confirming that this sequence corresponded to the amyloid fibril precursor deposited in the kidney.

A comprehensive investigation of amyloidogenic light chains should extend beyond primary structure to include complex PTMs. Previous studies identified N-glycosylated monoclonal light chains primarily by detecting the characteristic molecular mass shift before and after PNGase F treatment using mass spectrometry or gel electrophoresis, establishing N-glycosylated monoclonal light chains as a characteristic molecular feature of AL amyloidosis (9, 11). However, the biological significance of this modification remains largely unresolved. Increasing evidence has characterized the distribution of N-glycosylation sites in AL amyloidosis. They are typically acquired during somatic hypermutation, exhibit a marked germline bias (e.g., *IGKV1-16, IGKV1-33, IGKV1-39, IGKV4-1, IGLV1-51,* and *IGLV2-14*), and preferentially localize to recurrent hotspots within the CDRs and FR3 regions (33, 44). Nevertheless, these hotspots have largely been predicted from light-chain sequences, leaving unanswered whether the predicted N-glycosylation sequons are occupied by glycans and whether the resulting N-glycosylated light chains are incorporated into tissue amyloid fibrils. To address these questions, urinary light chains were compared before and after PNGase F treatment and then examined in matched renal amyloid deposits. Peptides spanning the predicted N-glycosylation sequons were detected after deglycosylation and were predominantly deamidated at Asn (+0.98 Da), supporting N-glycosylation at these sites. The same deamidated asparagine was identified in renal deposits, supporting the incorporation of N-glycosylated light chains into amyloid fibrils. As highlighted by the International Myeloma Working Group Mass Spectrometry Committee, further studies are required to clarify the role of N-glycosylation in AL amyloidosis (45). Our workflow provides a practical platform for addressing these unresolved mechanistic questions.

There are technical limitations in this study. First, V_L_ coverage differed among renal amyloid samples. In specimens with sparse amyloid deposits, the low abundance of light-chain peptides relative to tissue proteins, together with background keratin contamination, limited peptide detection. Second, *de novo* sequencing cannot unambiguously distinguish Leu from Ile because these residues are isobaric. Accordingly, L/I assignments were inferred from the cleavage preferences of chymotrypsin and pepsin, although definitive isomer discrimination remains beyond the resolving power of the method.

In summary, we implemented an integrated analytical workflow using bottom-up *de novo* sequencing to assemble full-length amyloidogenic light-chain sequences in AL amyloidosis. Sequence agreement with matched renal amyloid deposits supports urinary light-chain analysis as a non-invasive alternative to bone marrow aspiration for obtaining amyloidogenic light-chain sequences. In addition, this workflow enabled sequencing of N-glycosylated light chains by overcoming glycosylation-associated sequence gaps, providing a practical framework for mechanistic studies of amyloidogenic light chains and other light-chain–related diseases.

## Data Availability

The mass spectrometry proteomics data have been deposited to the ProteomeXchange Consortium via the iProX partner repository under the dataset identifier PXD077287.

## Supplemental Data

This article contains supplemental data.

## Conflict of Interest

The authors declare that they do not have any conflicts of interest with the content of this article.
