# Supplementary material for: Mass Spectrometry-Based De Novo Sequencing of N-Glycosylated Light Chains: Linking Urinary Proteoforms to In Situ Renal Amyloid Deposits
Source: Mol Cell Proteomics. 2026 Sep 1;25(10):101652. doi: 10.1016/j.mcpro.2026.101652 (PMC13635717; doi:10.1016/j.mcpro.2026.101652)
Supplement: Supplementary file 1 [file mmc1.pdf]

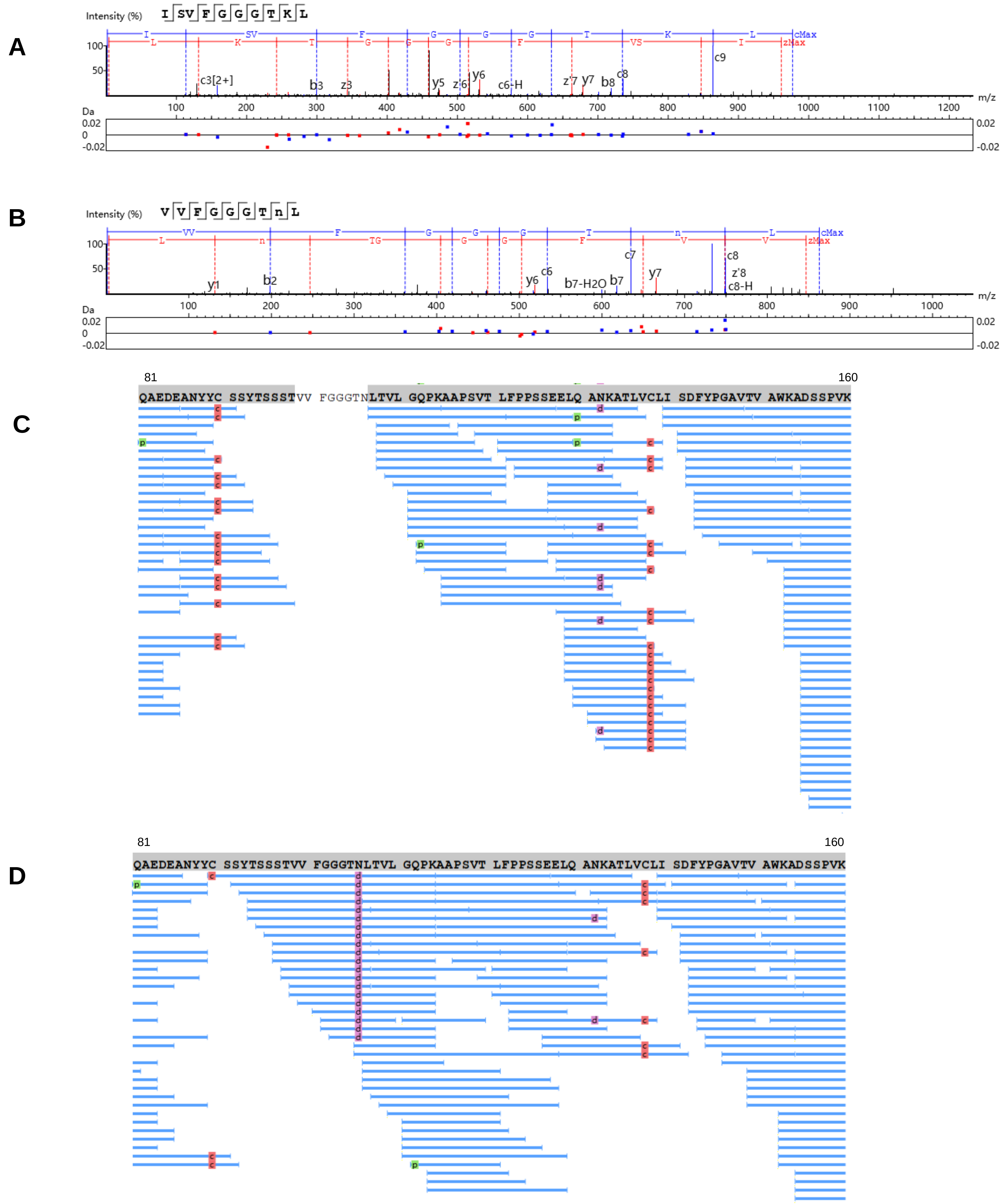

Supplementary Figure S1. MS/MS spectra and integrated peptide mapping of the PtC1-λ N-glycosylation region before and after PNGase F treatment.

(A) Representative MS/MS spectrum from the untreated sample showing a peptide derived from a background protein (ISVFGGGTKL). (B) Following PNGase F treatment, the PtC1-λ peptide VVFGGGTnL was identified, matching the assembled sequence and supporting localization of the N-glycosylation site. The lowercase “n” denotes a deamidated asparagine. (C, D) Enlarged peptide-coverage maps of PtC1-λ residues 81–160 before (C) and after (D) PNGase F treatment. Before treatment, no PtC1-λ peptide spanning the N-glycosylation site was detected, whereas deamidated site-spanning peptides were detected after treatment. Horizontal blue bars indicate supporting peptides identified by integrated database searching across the complementary protease digests performed for each condition. The purple “d” denotes a deamidated asparagine.

```
                    FR1                      CDR1          FR2       CDR2               FR3                   CDR3      FR4
PtL1-κ DIQMTQSPSSLSASVGDGVTLTCRASQGI------SNDLAWFQQRPGQAPKSLLYAA-------SSLQSGVP-SRFTGSG--SGTDFTLTISRLQPEDFATYYCQQYNT----YPITFGQGTRLEIN IGKV1-16_IGKJ5
PtZ1-κ DIQMTQSPSSLSASVGDRVTITCQASQDI------STSLNWYQQSPGKAPKLLIYAA-------SNLETGVP-SRFSGSG--SGTNFSVTISSLQPEDFARFYCQQFHD----LPFTFGGGTMVETK IGKV1-33_IGKJ4
PtZ2-κ DIQMTQSPSSLSASVGDRVTITCQASQDI------SYYLNWYQQKSGKAPEFLIYDA-------SNLDAGVP-SRFSGNG--SGTHFTFTISSLQPADIATYYCQQYDN----LPYTFGQGTKLEIK IGKV1-33_IGKJ2
PtH1-κ DIQMTQSPSSLSASLGDSVTLTCRANQTI------SNYLNWYQQTPGKAPEVLIYAA-------SILYRGVS-SRFSGSG--SGTDFTLTLSSIQPEDFAAYFCHQSHS----IPWTFGQGTTVEFK IGKV1-39_IGKJ1
PtB1-κ VIQMTQSPSSLSASIGGRFTITCRASQNI------STFLNWYQQKAGKAPNLLIYGA-------SSLQSGVP-SRFSGSG--SGTDFTLTINSLQPEDFATYYCQQSYN----NPLSFGPGTKVDMK IGKV1-39_IGKJ3
PtB2-κ DIVMTQSPDSLAVSLGERATINCKSSQSVSYTSNNKTYLAWHQQKPGQPPKVVIYWA-------STRESGVP-DRFSGSG--SGTDFSLTISSIQAEDVAVYYCQQYYN----FPQTFGGGTKVEIK IGKV4-1_IGJK4
PtC1-λ QSALTQPAS-VSGSPGQSISISCTGTSSDVG---LYNIVSWYQQHPGTAPKLLIYEV-------YNRPSGVS-NRFSGSK--SGNTASLTISGLQAEDEANYYCSSYTS---SSTVVFGGGTNLTVL IGLV2-14_IGLJ2
PtT1-λ QSALTQPAS-VAGSPGQSITLSCTGSSSDIG---NYKYVSWYQQYPGKAPKLLIYDI-------SERPSGLS-SRFSGSK--SGNTASLTISGLQAEDEADFYCSSYTS---NNTYVFGGGTRLTVL IGLV2-14_IGLJ3
```

```
                                              C-region
PtL1-κ RTVAAPSVFIFPPSDEQLKSGTASVVCLLNNFYPREAKVQWKVDNALQSGNSQESVTEQDSKDSTYSLSSTLTLSKADYEKHKLYACEVTHQGLSSPVTKSFNRGEC IGKC*04
PtZ1-κ RTVAAPSVFIFPPSDEQLKSGTASVVCLLNNFYPREAKVQWKVDNALQSGNSQESVTEQDSKDSTYSLSSTLTLSKADYEKHKLYACEVTHQGLSSPVTKSFNRGEC IGKC*04
PtZ2-κ RTVAAPSVFIFPPSDEQLKSGTASVVCLLNNFYPREAKVQWKVDNALQSGNSQESVTEQDSKDSTYSLSSTLTLSKADYEKHKLYACEVTHQGLSSPVTKSFNRGEC IGKC*04
PtH1-κ RTVAAPSVFIFPPSDEQLKSGTASVVCLLNNFYPREAKVQWKVDNALQSGNSQESVTEQDSKDSTYSLSSTLTLSKADYEKHKVYACEVTHQGLSSPVTKSFNRGEC IGKC*01
PtB1-κ RTVAAPSVFIFPPSDEQLKSGTASVVCLLNNFYPREAKVQWKVDNALQSGNSQESVTEQDSKDSTYSLSSTLTLSKADYEKHKLYACEVTHQGLSSPVTKSFNRGEC IGKC*04
PtB2-κ RTVAAPSVFIFPPSDEQLKSGTASVVCLLNNFYPREAKVQWKVDNALQSGNSQESVTEQDSKDSTYSLSSTLTLSKADYEKHKLYACEVTHQGLSSPVTKSFNRGEC IGKC*04
PtC1-λ GQPKAAPSVTLFPPSSEELQANKATLVCLISDFYPGAVTVAWKADSSPVKAGVETTTPSKQSNNKYAASSYLSLTPEQWKSHRSYSCQVTHEGSTVEKTVAPTECS-  IGLC2
PtT1-λ GQPKAAPSVTLFPPSSEELQANKATLVCLISDFYPGAVTVAWKADSSPVKAGVETTTPSKQSNNKYAASSYLSLTPEQWKSHKSYSCQVTHEGSTVEKTVAPTECS-  IGLC3
```

Supplementary Figure S2. Alignment of the assembled full-length amino acid sequences of the eight monoclonal light chains.

The V_L and C_L domains are shown separately. The V_L sequences were aligned according to the standardized numbering scheme of the international ImMunoGeneTics information system (IMGT), with FRs and CDRs indicated. The corresponding V-, J-, and constant-region gene assignments are shown at the right.

A

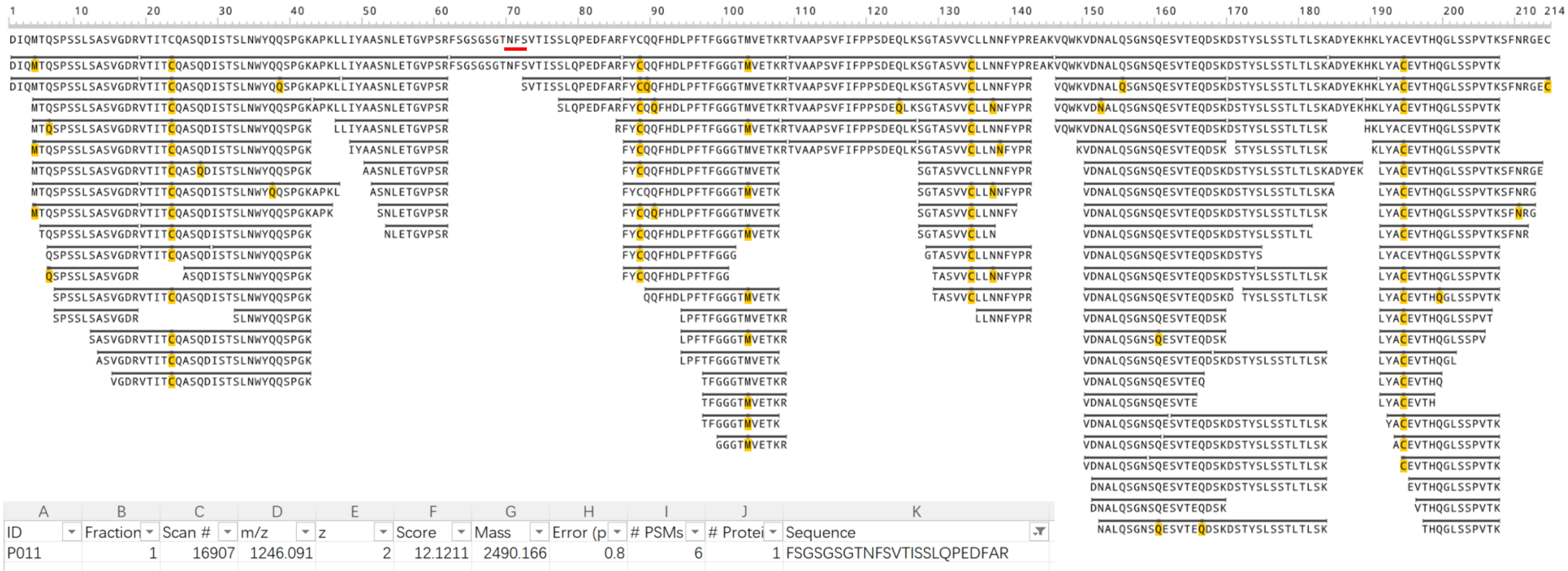

B

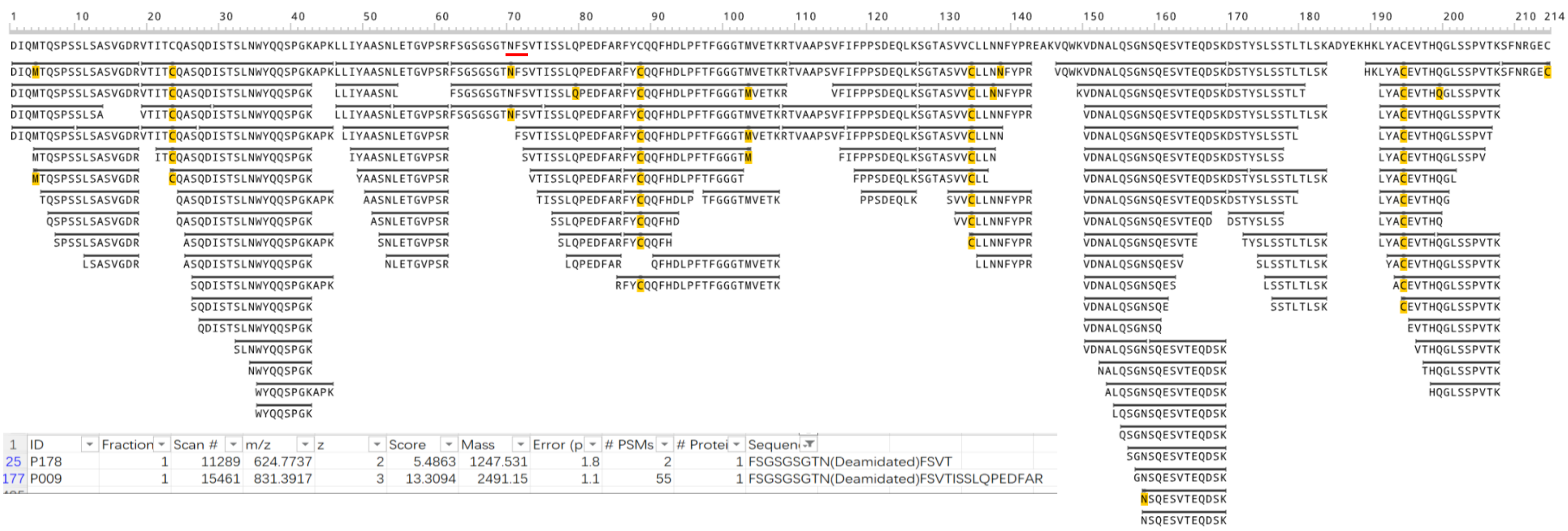

Supplementary Figure S3. Sequence coverage map of trypsin-digested PtZ1-κ.

(A) Without PNGase F treatment, the site-spanning peptide was detected only with the sequon Asn in its unmodified form, consistent with an unglycosylated fraction at this site.

(B) After PNGase F treatment, deamidated site-spanning peptides appeared or increased, consistent with PNGase F-mediated deglycosylation and supporting localization of the N-glycosylation site. Corresponding identified peptide-spectrum matches (PSMs) are listed below. The figures above are novor.cloud search results. The yellow-highlighted ‘N’ represents a deamidation asparagine. Red underlining indicates the N-glycosylation site.

A

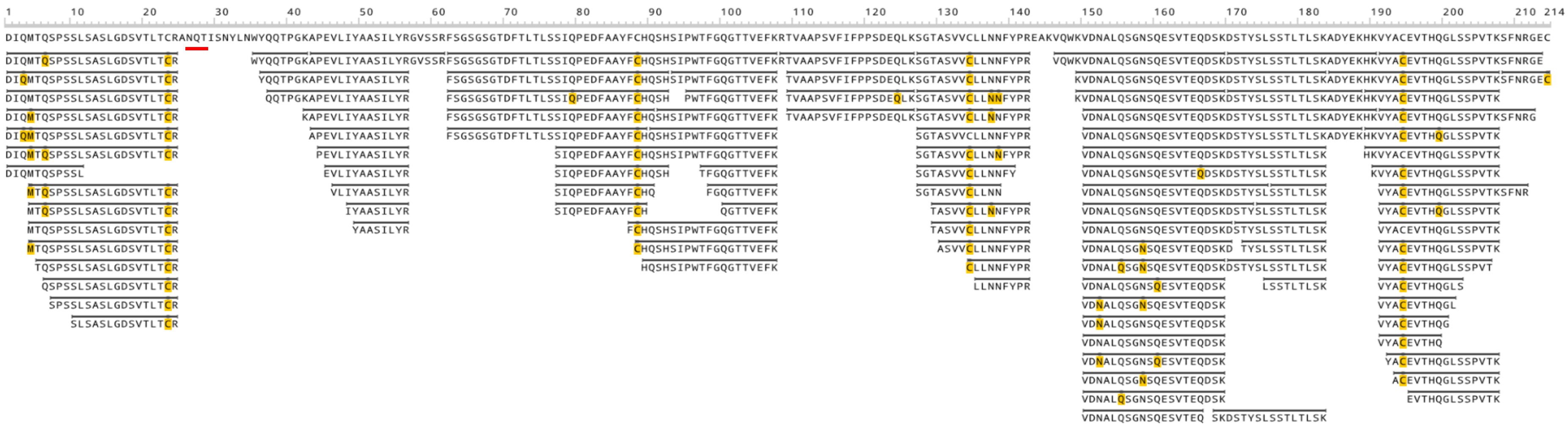

B

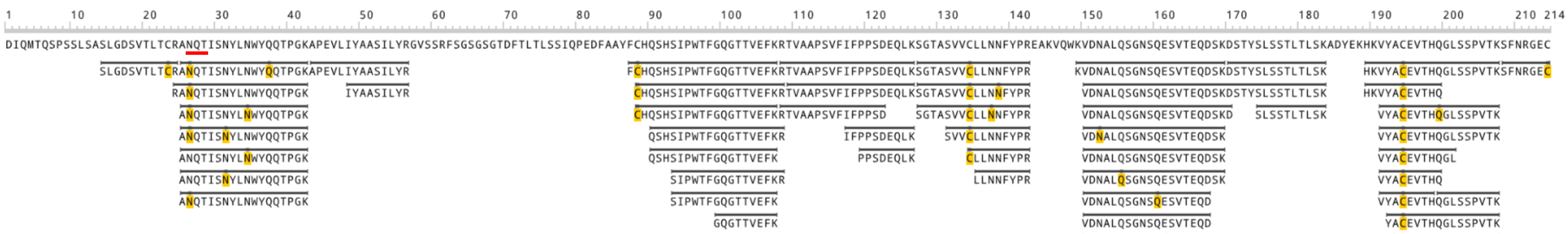

	A	B	C	D	E	F	G	H	I	J	K
1	ID	Fraction	Scan #	m/z	z	Score	Mass	Error (ppm	# PSMs	# Proteins	Sequence
6	P105	1	9096	710.0067	3	6.9483	2126.991	3.4	1	1	AN(Deamidated)QTISNYLNWYQ(Deamidated)QTPGK
52	P071	1	8593	710.0046	3	8.5297	2126.991	0.5	1	1	AN(Deamidated)QTISNYLN(Deamidated)WYQQTPGK
75	P087	1	9178	1064.506	2	7.4703	2126.991	3.4	1	1	AN(Deamidated)QTISN(Deamidated)YLNWYQQTPGK
178	P044	1	9129	1064.01	2	9.9803	2126.007	-0.3	23	1	AN(Deamidated)QTISNYLNWYQQTPGK

Supplementary Figure S4. Sequence coverage map of trypsin-digested PtH1-κ.

(A) Without PNGase F treatment: Peptides spanning the N-glycosylation site were undetected.

(B) After PNGase F treatment, deamidated site-spanning peptides appeared or increased, consistent with PNGase F-mediated deglycosylation and supporting localization of the N-glycosylation site. Corresponding identified peptide-spectrum matches (PSMs) are listed below. The figures above are novor.cloud search results. The yellow-highlighted ‘N’ represents a deamidation asparagine. Red underlining indicates the N-glycosylation site.

A

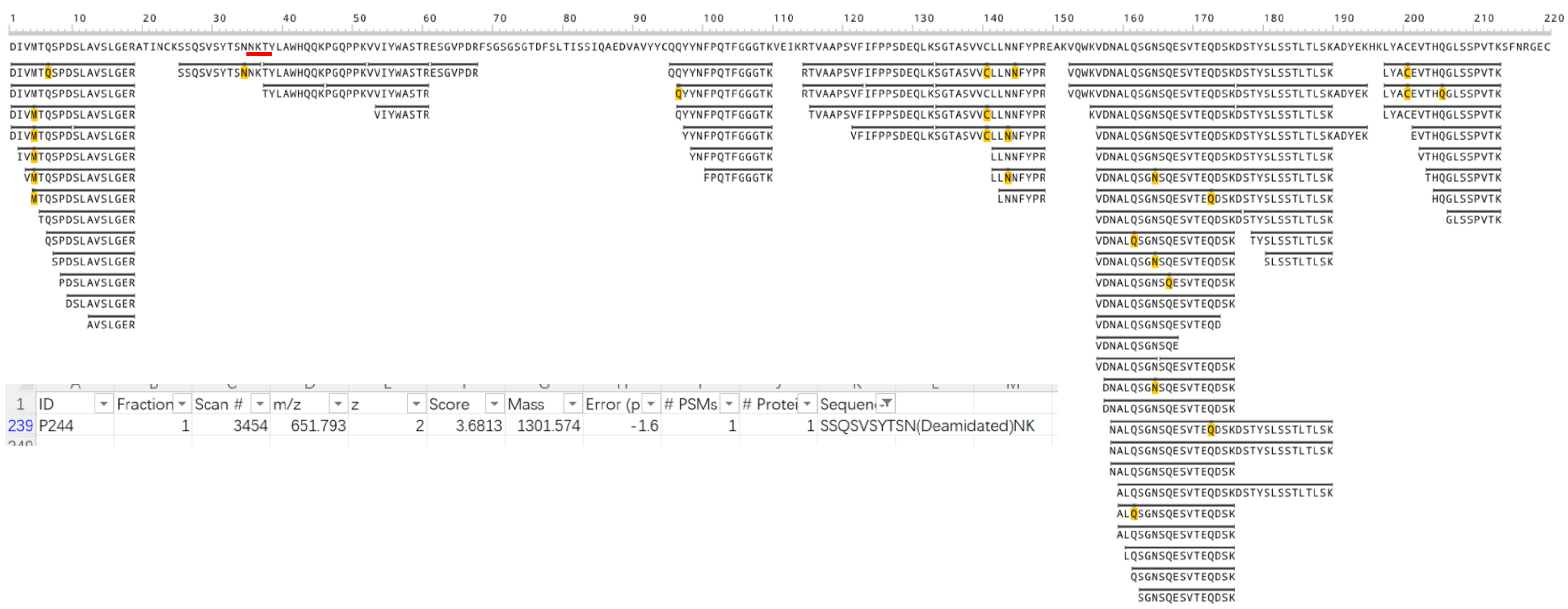

	ID	Fraction	Scan #	m/z	z	Score	Mass	Error (p	# PSMs	# Protei	Sequence
239	P244	1	3454	651.793	2	3.6813	1301.574	-1.6	1	1	SSQSVSYTSN(Deamidated)NK

B

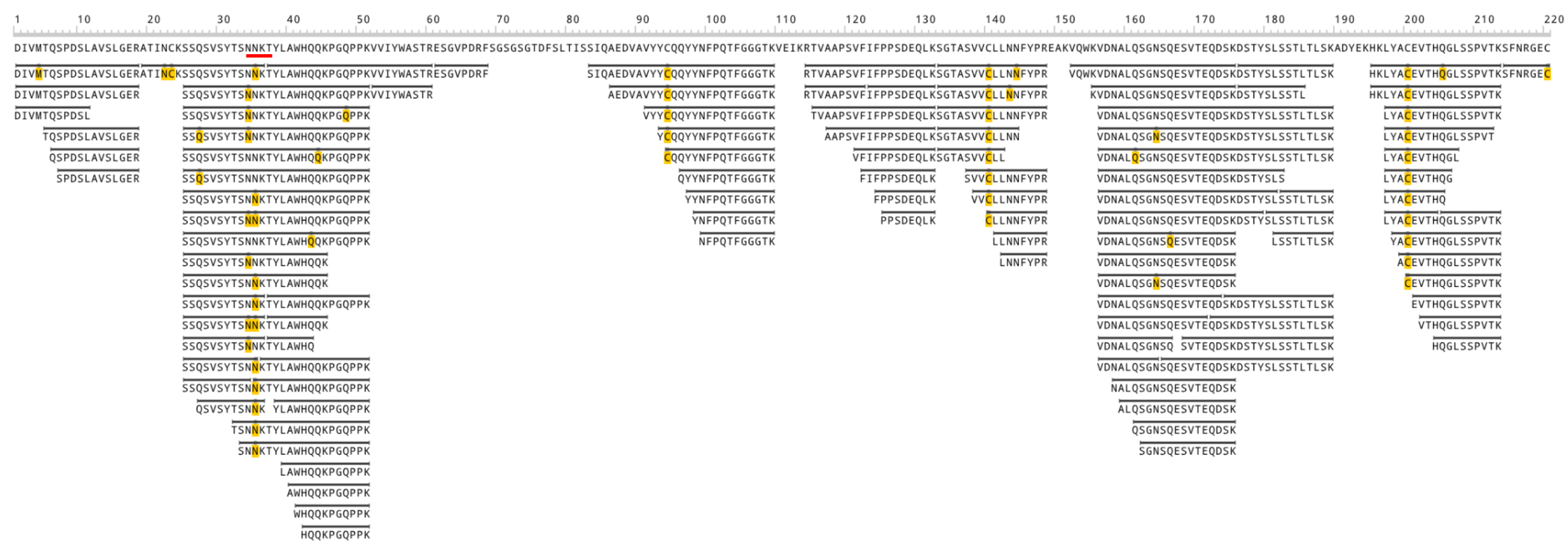

	ID	Fraction	Scan #	m/z	z	Score	Mass	Error (p	# PSMs	# Protei	Sequence
13	P123	1	6505	664.3082	3	8.279	1989.895	3.9	4	1	ATIN(Deamidated)C(Cam)KSSQSVSYTSNN(Deamidated)K
19	P082	1	7862	506.2698	4	10.2004	2021.048	0.9	4	1	N(Deamidated)KTYLAWHQQKPGQPPK
58	P175	1	5290	651.7953	2	6.7345	1301.574	1.9	19	1	SSQSVSYTSNN(Deamidated)K
158	P023	1	7730	581.8009	4	13.1156	2323.171	1.5	6	1	TSNN(Deamidated)KTYLAWHQQKPGQPPK
267	P247	1	7744	556.5388	4	5.0279	2222.123	1.3	2	1	SNN(Deamidated)KTYLAWHQQKPGQPPK
350	P205	1	5483	652.2889	2	5.7803	1302.558	4.4	4	1	SSQSVSYTSN(Deamidated)N(Deamidated)K
353	P002	1	8858	766.382	4	19.6551	3061.489	3.1	22	1	SSQSVSYTSNN(Deamidated)KTYLAWHQQKPGQPPK
368	P003	1	9122	766.6268	4	18.9102	3062.473	1.5	17	1	SSQSVSYTSN(Deamidated)N(Deamidated)KTYLAWHQQKPGQPPK
470	P080	1	9389	615.2969	4	10.2626	2457.156	0.9	2	1	SSQSVSYTSNN(Deamidated)KTYLAWHQQK
489	P307	1	5238	564.7639	2	4.1033	1127.51	3.4	1	1	QSVSYTSNN(Deamidated)K

Supplementary Figure S5. Sequence coverage map of trypsin-digested PtB2-κ.

(A) Without PNGase F treatment, the N-glycosylation-site Asn was detected only in its unmodified form, consistent with an unglycosylated fraction at this site.

(B) After PNGase F treatment, deamidated site-spanning peptides appeared or increased, consistent with PNGase F-mediated deglycosylation and supporting localization of the N-glycosylation site. Corresponding identified peptide-spectrum matches (PSMs) are listed below. The figures above are novor.cloud search results. The yellow-highlighted ‘N’ represents a deamidation asparagine. Red underlining indicates the N-glycosylation site.

A

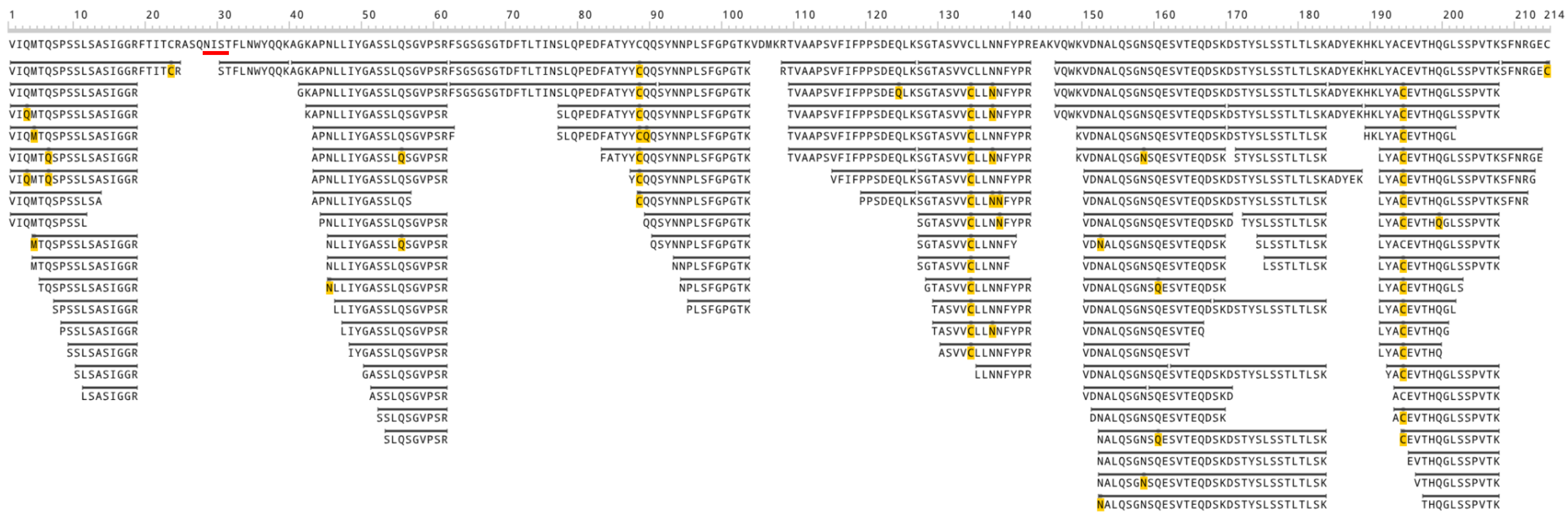

B

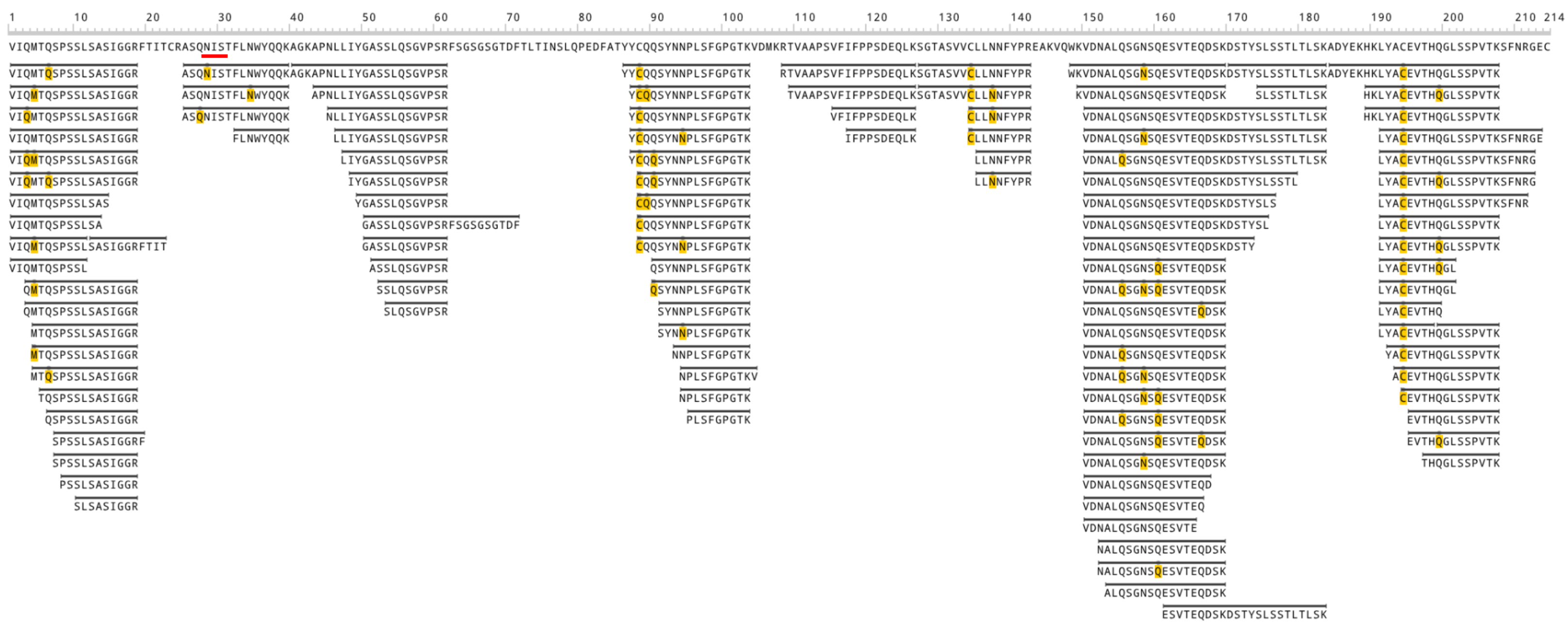

	A	B	C	D	E	F	G	H	I	J	K
1	ID	Fraction	Scan #	m/z	z	Score	Mass	Error (p	# PSMs	# Protei	Sequence
80	P105	1	13081	914.9465	2	9.9775	1827.879	-0.4	8	1	ASQN(Deamidated)ISTFLNWYQQK
302											

Supplementary Figure S6. Sequence coverage map of trypsin-digested PtB1-κ.

(A) Without PNGase F treatment: Peptides spanning the N-glycosylation site were undetected.

(B) After PNGase F treatment, deamidated site-spanning peptides appeared or increased, consistent with PNGase F-mediated deglycosylation and supporting localization of the N-glycosylation site. Corresponding identified peptide-spectrum matches (PSMs) are listed below. The figures above are novor.cloud search results. The yellow-highlighted 'N' represents a deamidation asparagine. Red underlining indicates the N-glycosylation site.

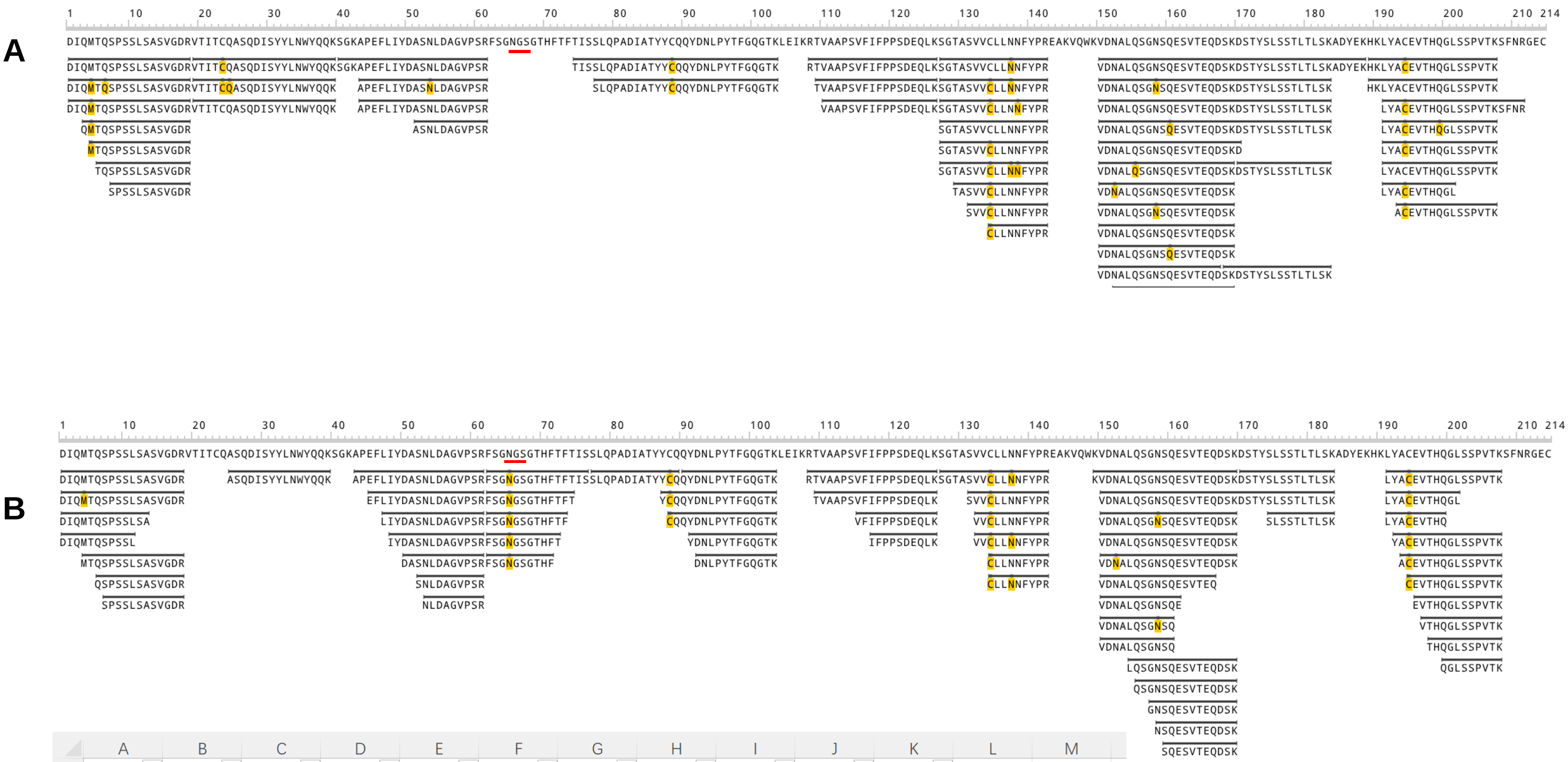

	ID	Fraction	Scan #	m/z	z	Score	Mass	Error (p	# PSMs	# Protei	Sequen
47	P293	1	9318	630.2687	2	5.8973	1258.526	-2	1	1	FSGN(Deamidated)GSGTHFTF
124	P545	1	7099	506.2132	2	3.6888	1010.409	2.4	1	2	FSGN(Deamidated)GSGTHF
265	P280	1	6795	556.7354	2	6.0918	1111.457	-0.8	1	2	FSGN(Deamidated)GSGTHFT
442	P166	1	8845	680.7933	2	7.6514	1359.573	-0.8	1	1	FSGN(Deamidated)GSGTHFTFT
466	P407	1	9911	780.8513	2	4.7585	1559.689	-0.8	1	1	FSGN(Deamidated)GSGTHFTFTIS

Supplementary Figure S7. Sequence coverage map of trypsin-digested PtZ2-κ.

(A) Without PNGase F treatment, peptides spanning the N-glycosylation site were not detected.

(B) After PNGase F treatment, deamidated site-spanning peptides appeared or increased, consistent with PNGase F-mediated deglycosylation and supporting localization of the N-glycosylation site. Corresponding identified peptide-spectrum matches (PSMs) are listed below. The figures above are novor.cloud search results. The yellow-highlighted ‘N’ represents a deamidation asparagine. Red underlining indicates the N-glycosylation site.

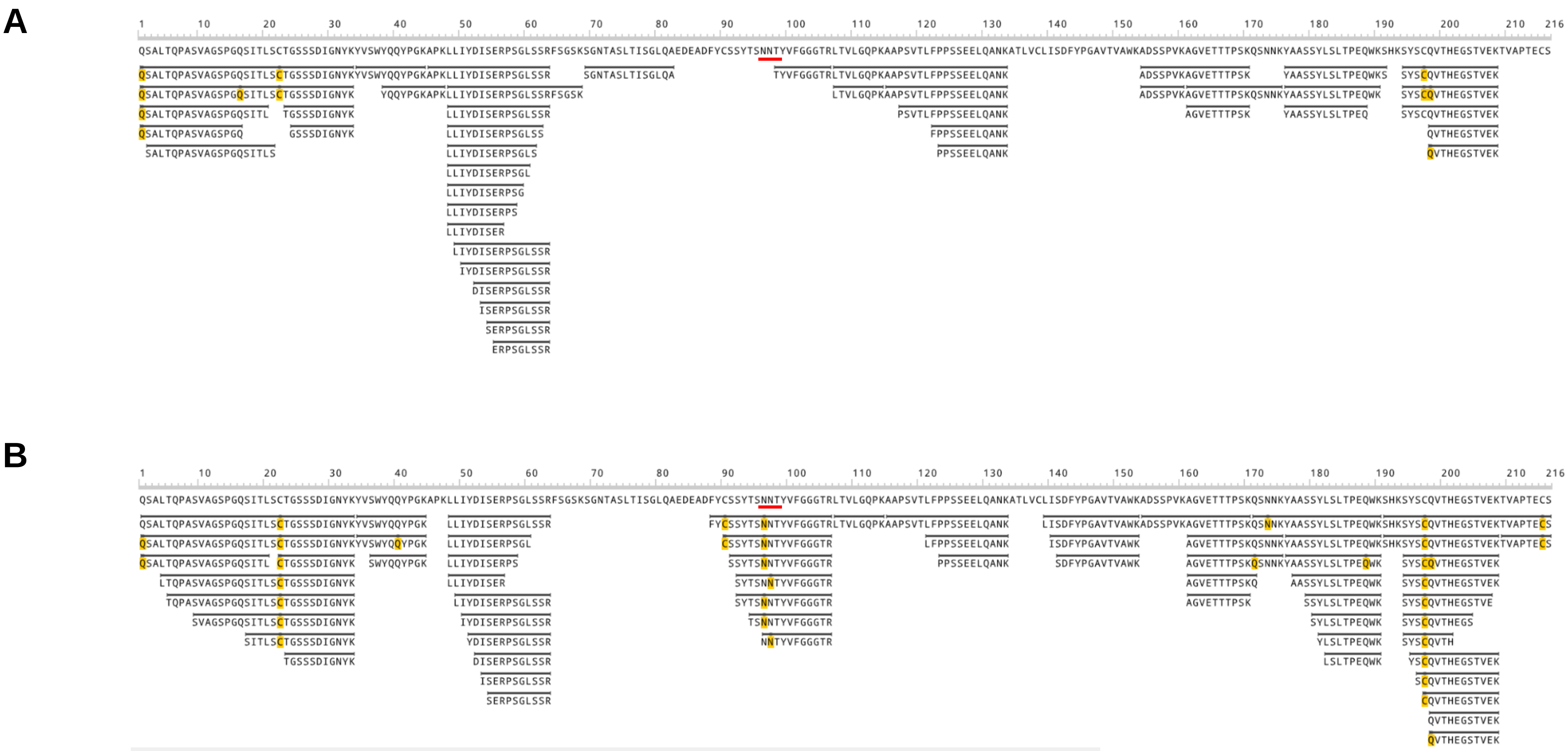

	ID	Fraction	Scan #	m/z	z	Score	Mass	Error (p	# PSMs	# Protei	Sequence
66	P037	1	7854	1091.462	2	13.2898	2180.911	-1	2	1	FYC(Cam)SSYTSN(Deamidated)NTYVFGGGTR
153	P157	1	6651	687.8173	2	7.3027	1373.621	-0.9	1	1	TSN(Deamidated)NTYVFGGGTR
184	P061	1	7045	856.3807	2	11.643	1710.749	-0.9	1	1	SSYTSN(Deamidated)NTYVFGGGTR
225	P078	1	7084	936.3961	2	10.9137	1870.779	-0.9	1	1	C(Cam)SSYTSN(Deamidated)NTYVFGGGTR
268	P085	1	7062	812.8671	2	10.5185	1623.717	2	1	1	SYTSN(Deamidated)NTYVFGGGTR

Supplementary Figure S8. Sequence coverage map of trypsin-digested PtT1-λ.

(A) Without PNGase F treatment: Peptides spanning the N-glycosylation site were undetected.

(B) After PNGase F treatment, deamidated site-spanning peptides appeared or increased, consistent with PNGase F-mediated deglycosylation and supporting localization of the N-glycosylation site. Corresponding identified peptide-spectrum matches (PSMs) are listed below. The figures above are novor.cloud search results. The yellow-highlighted ‘N’ represents a deamidation asparagine. Red underlining indicates the N-glycosylation site.

A

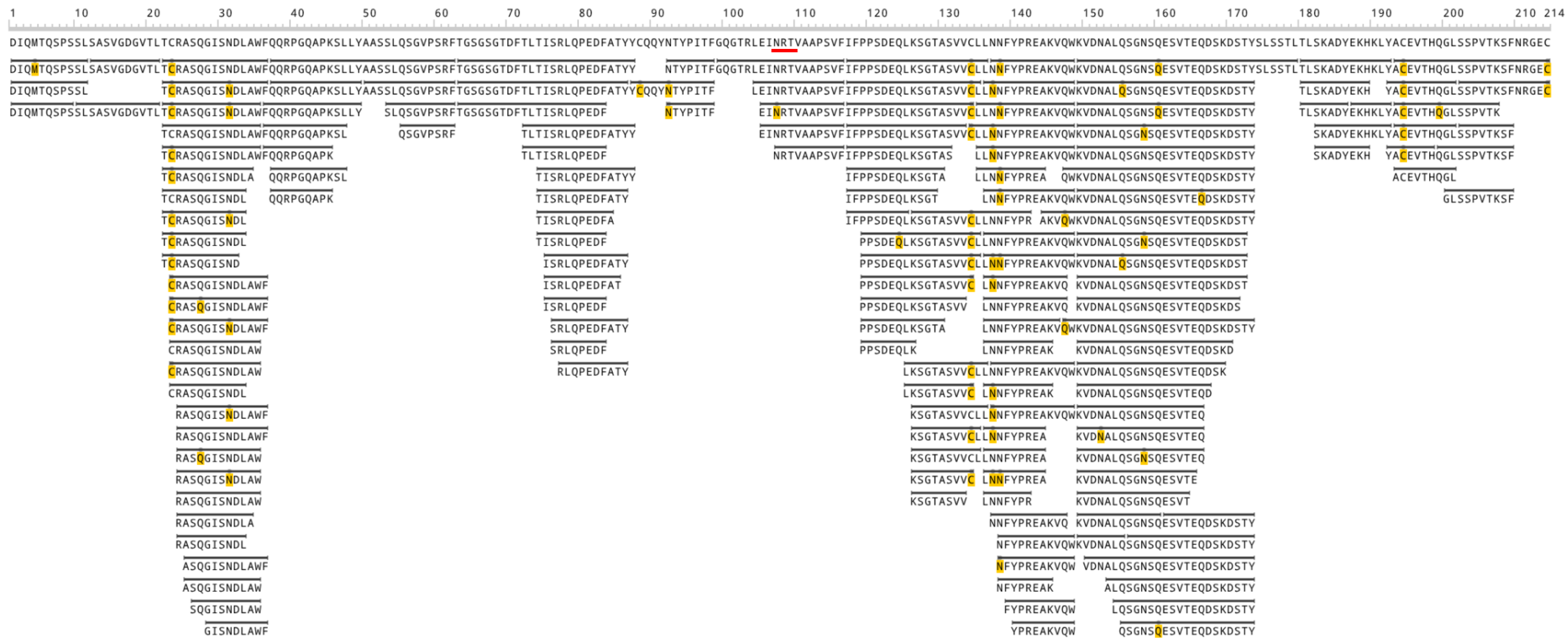

	ID	Fraction	Scan #	m/z	z	Score	Mass	Error (p	# PSMs	# Protei	Sequence
22	P157	1	14249	958.515	2	3.0632	1915.028	-6.4	2	2	GQGTRLEINRTVAAPSVF
34	P190	1	13276	652.8414	2	2.7676	1303.677	-6.8	1	2	EIN(Deamidated)RTVAAPSVF
167	P224	1	10482	531.2866	2	2.4738	1060.567	-7.5	1	5	NRTVAAPSVF
226	P144	1	16384	708.8909	2	3.2856	1415.777	-7	1	2	LEINRTVAAPSVF
250	P036	1	21173	652.3502	2	5.5168	1302.693	-5.7	63	2	EINRTVAAPSVF

B

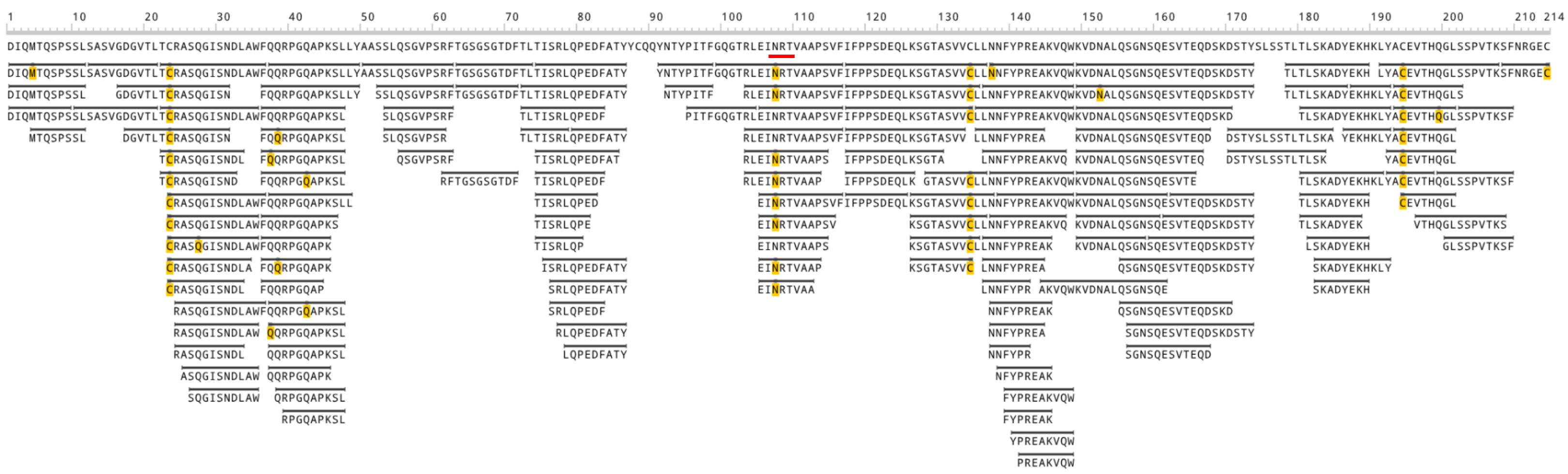

	ID	Fraction	Scan #	m/z	z	Score	Mass	Error (ppm	# PSMs	# Proteins	Sequence
10	P152	1	6352	579.312	2	4.769	1156.609	0.5	1	1	EIN(Deamidated)RTVAAPSV
46	P043	1	7740	787.439	2	9.5233	1572.862	0.7	2	1	RLEIN(Deamidated)RTVAAPSVF
57	P205	1	5040	437.7361	2	3.3257	873.4556	2.4	1	1	EIN(Deamidated)RTVAA
73	P129	1	6104	443.2498	3	5.4385	1326.726	1.5	2	1	RLEIN(Deamidated)RTVAAPS
80	P017	1	7749	959.0136	2	12.0289	1916.012	0.5	2	1	GQGTRLEIN(Deamidated)RTVAAPSVF
121	P214	1	5670	486.2618	2	3.0616	970.5084	0.7	1	1	EIN(Deamidated)RTVAAP
181	P071	1	6375	620.8547	2	7.5928	1239.694	1.1	2	1	RLEIN(Deamidated)RTVAAP
216	P042	1	7610	652.8486	2	9.5854	1303.677	4.2	38	1	EIN(Deamidated)RTVAAPSVF

Supplementary Figure S9. Sequence coverage map of chymotrypsin-digested PtL1-κ.

(A) Without PNGase F treatment, site-spanning peptides were detected predominantly with the sequon Asn unmodified, although a low-abundance deamidated form was also observed.

(B) After PNGase F treatment, deamidated site-spanning peptides appeared or increased, consistent with PNGase F-mediated deglycosylation and supporting localization of the N-glycosylation site. Corresponding identified peptide-spectrum matches (PSMs) are listed below. The figures above are novor.cloud search results. The yellow-highlighted 'N' represents a deamidation asparagine. Red underlining indicates the N-glycosylation site.

ID	Score	Peptides	PSMs	% Covered	Length	MW	Description
G001-01	6180	838	3664	100.0	214	23.3	PtL1-κ_IGKV1-16_IGKJ5_IGKC4
G001-02	97	16	59	37.9	95	10.41	X64133|IGKV1D-33*01|HOMO SAPIENS|V-REGION
G001-03	97	19	38	14.8	81	8.91	AF490932|IGKV3-15*01|HOMO SAPIENS|V-REGION
G001-04	83	14	57	30.8	117	12.85	SP|P01593-LDY-27|KVD33_HUMAN IMMUNOGLOBULIN KAPPA VARIABLE 1D-33 OS=HOMO SAPIENS OX=9606 GN=IGKV1D-33 PE=1 SV=2
G001-05	78	13	71	34.0	97	10.57	P01599|IGKV1-17*02|HOMO SAPIENS|V-REGION

>PtL1-κ_IGKV1-16_IGKJ5_IGKC4
DIQMTQSPSSLSASVGDGVTLTCRASQGISNDLAWFQQRPGQAPKSLLYAASSLQSGVPSRFTGSGSGTDFTLTISRLQPEDFATYYCQQYNTYPITFGQ
GTRLEINRTVAAPSVFIFPPSDEQLKSGTASVVCLLNNFYPREAKVQWKVDNALQSGNSQESVTEQDSKDSTYSLSSTLTLSKADYEKHKLYACEVTHQG
LSSPVTKSFNRGEC

ID	Score	Peptides	PSMs	% Covered	Length	MW	Description
G001-001	6090	905	7355	100.0	214	23.27	PtZ1-κ_IGKV1-33_IGKJ4_IGKC4
G001-002	3584	519	4762	100.0	107	11.78	SP|P01834-LDY-4|IGKC_HUMAN IMMUNOGLOBULIN KAPPA CONSTANT OS=HOMO SAPIENS OX=9606 GN=IGKC PE=1 SV=2
G001-003	2963	445	3843	100.0	107	11.78	AF017732|IGKC*04|HOMO SAPIENS|F|C-REGION|C,62461..62780|321 NT|1|+1| | |107 AA|107+0=107| | |
G001-004	2963	445	3843	100.0	107	11.78	EF589488|IGKC*04|HOMO SAPIENS|C-REGION
G001-005	2412	356	3097	77.6	107	11.9	AY900167|IGKC*01|HOMO SAPIENS|C-REGION

>PtZ1-κ_IGKV1-33_IGKJ4_IGKC4
DIQMTQSPSSLSASVGDRVTITCQASQDISTSLNWYQQSPGKAPKLLIYAASNLETGVPSRFSGSGSGTNFSVTISSLQPEDFARFYCQQFHDLPFTFGG
GTMVETKRTVAAPSVFIFPPSDEQLKSGTASVVCLLNNFYPREAKVQWKVDNALQSGNSQESVTEQDSKDSTYSLSSTLTLSKADYEKHKLYACEVTHQG
LSSPVTKSFNRGEC

ID	Score	Peptides	PSMs	% Covered	Length	MW	Description
G0001-01	3014	441	1532	100.0	214	23.56	PtZ2-κ_IGKV1-33_IGKJ2_IGKC4
G0001-02	220	30	128	47.9	96	10.54	SP|P01593-LDY-44|KVD33_HUMAN IMMUNOGLOBULIN KAPPA VARIABLE 1D-33 OS=HOMO SAPIENS OX=9606 GN=IGKV1D-33 PE=1 SV=2
G0001-03	216	29	127	40.0	95	10.39	SP|P01593-LDY-42|KVD33_HUMAN IMMUNOGLOBULIN KAPPA VARIABLE 1D-33 OS=HOMO SAPIENS OX=9606 GN=IGKV1D-33 PE=1 SV=2
G0001-04	191	27	112	32.5	117	12.85	SP|P01593-LDY-8|KVD33_HUMAN IMMUNOGLOBULIN KAPPA VARIABLE 1D-33 OS=HOMO SAPIENS OX=9606 GN=IGKV1D-33 PE=1 SV=2
G0001-05	184	25	113	50.0	98	10.77	EU599323|IGKV1D-33*01|HOMO SAPIENS|V-REGION

>PtZ2-κ_IGKV1-33_IGKJ2_IGKC4
DIQMTQSPSSLSASVGDRVTITCQASQDISYYLNWYQQKSGKAPEFLIYDASNLDAGVPSRFSGNGSGTHFTFTISSLQPADIATYYCQQYDNLPYTFGQ
GTKLEIKRTVAAPSVFIFPPSDEQLKSGTASVVCLLNNFYPREAKVQWKVDNALQSGNSQESVTEQDSKDSTYSLSSTLTLSKADYEKHKLYACEVTHQG
LSSPVTKSFNRGEC

ID	Score	Peptides	PSMs	% Covered	Length	MW	Description
G001-01	4396	653	1683	100.0	214	23.4	PtH1-κ_IGKV1-39_IGKJ1_IGKC1
G001-02	2611	373	1012	98.1	106	11.64	SP|P01834-LDY-6|IGKC_HUMAN IMMUNOGLOBULIN KAPPA CONSTANT OS=HOMO SAPIENS OX=9606 (
G001-03	71	14	18	22.7	97	10.42	AY867406|IGKV1D-39*01|HOMO SAPIENS|V-REGION
G001-04	59	10	18	24.7	97	10.38	EF589452|IGKV1D-12*02|HOMO SAPIENS|V-REGION
G001-05	52	9	17	22.9	96	10.5	P01596|IGKV1-5*03|HOMO SAPIENS|V-REGION

>PtH1-κ_IGKV1-39_IGKJ1_IGKC1
DIQMTQSPSSLSASLGDSVTLTCRANQTISNYLNWYQQTPGKAPEVLIYAASILYRGVSSRFSGSGSGTDFTLTLSSIQPEDFAAYFCHQSHSIPWTFGQ
GTTVEFKRTVAAPSVFIFPPSDEQLKSGTASVVCLLNNFYPREAKVQWKVDNALQSGNSQESVTEQDSKDSTYSLSSTLTLSKADYEKHKVYACEVTHQG
LSSPVTKSFNRGEC

ID	Score	Peptides	PSMs	% Covered	Length	MW	Description
G001-01	4154	543	2300	100.0	214	23.28	PtB1-κ_IGKV1-39_IGKJ3_IGKC4
G001-02	2252	292	1517	100.0	107	11.78	EF589488|IGKC*04|HOMO SAPIENS|C-REGION
G001-03	2252	292	1517	100.0	107	11.78	AF017732|IGKC*04|HOMO SAPIENS|F|C-REGION|C,62461..62780|321 NT|1|+1| | |107 AA|107+0=107| | |
G001-04	2037	271	1424	100.0	107	11.78	SP|P01834-LDY-4|IGKC_HUMAN IMMUNOGLOBULIN KAPPA CONSTANT OS=HOMO SAPIENS OX=9606 GN=IGKC PE=1 SV=2
G001-05	1531	204	1320	74.8	107	11.9	AY900167|IGKC*01|HOMO SAPIENS|C-REGION

>PtB1-κ_IGKV1-39_IGKJ3_IGKC4
VIQMTQSPSSLSASIGGRFTITCRASQNISTFLNWYQQKAGKAPNLLIYGASSLQSGVPSRFSGSGSGTDFTLTINSLQPEDFATYYCQQSYNNPLSFGP
GTKVDMKRTVAAPSVFIFPPSDEQLKSGTASVVCLLNNFYPREAKVQWKVDNALQSGNSQESVTEQDSKDSTYSLSSTLTLSKADYEKHKLYACEVTHQG
LSSPVTKSFNRGEC

ID	Score	Peptides	PSMs	% Covered	Length	MW	Description
G001-01	5603	728	6458	100.0	220	24.15	PtB2-κ_IGKV4-1_IGKJ4_IGKC*04
G001-02	104	16	92	53.3	60	6.6	SP|P06312-LDY-41|KV401_HUMAN IMMUNOGLOBULIN KAPPA VARIABLE 4-1 OS=HOMO SAPIENS
G001-03	102	16	91	48.3	60	6.79	SP|P06312-LDY-34|KV401_HUMAN IMMUNOGLOBULIN KAPPA VARIABLE 4-1 OS=HOMO SAPIENS
G001-04	102	16	91	48.3	60	6.65	SP|P06312-LDY-24|KV401_HUMAN IMMUNOGLOBULIN KAPPA VARIABLE 4-1 OS=HOMO SAPIENS
G001-05	100	15	90	40.0	60	6.67	SP|P06312-LDY-42|KV401_HUMAN IMMUNOGLOBULIN KAPPA VARIABLE 4-1 OS=HOMO SAPIENS

>PtB2-κ_IGKV4-1_IGKJ4_IGKC*04
DIVMTQSPDSLAVSLGERATINCKSSQSVSYTSNNKTYLAWHQQKPGQPPKVVIYWASTRESGVPDRFSGSGSGTDFSLTISSIQAEDVAVYYCQQYYNF
PQTFGGGTKVEIKRTVAAPSVFIFPPSDEQLKSGTASVVCLLNNFYPREAKVQWKVDNALQSGNSQESVTEQDSKDSTYSLSSTLTLSKADYEKHKLYAC
EVTHQGLSSPVTKSFNRGEC

ID	Score	Peptides	PSMs	% Covered	Length	MW	Description
G001-001	3101	465	3671	100.0	216	22.69	PtC1-λ_IGLV2-14_IGLJ2_IGLC2
G001-002	1472	222	2176	66.2	216	22.85	PtT1-λ_IGLV2-14_IGLJ3_IGLC3
G001-003	1275	186	2042	50.9	214	22.77	SP|WS-AL-9|WS-AL-9 OS=UNKNOWN
G001-004	1146	164	1732	97.2	106	11.32	EF589538|IGLC3*04|HOMO SAPIENS|C-REGION
G001-005	1134	160	1725	90.5	105	11.31	EU599358|IGLC2*02|HOMO SAPIENS|C-REGION

>PtC1-λ_IGLV2-14_IGLJ2_IGLC2
QSALTQPASVSGSPGQSISISCTGTSSDVGLYNIVSWYQQHPGTAPKLLIYEVYNRPSGVSNRFSGSKSGNTASLTISGLQAEDEANYYCSSYTSSSTVV
FGGGTNLTVLGQPKAAPSVTLFPPSSEELQANKATLVCLISDFYPGAVTVAWKADSSPVKAGVETTTPSKQSNNKYAASSYLSLTPEQWKSHRSYSCQVT
HEGSTVEKTVAPTECS

ID	Score	Peptides	PSMs	% Covered	Length	MW	Description
G001-01	3612	557	2029	100.0	216	22.85	PtT1-λ_IGLV2-14_IGLJ3_IGLC3
G001-02	2218	322	1082	99.1	106	11.31	EU599331|IGLC3*03|HOMO SAPIENS|C-REGION
G001-03	2206	318	1077	99.0	104	11.08	J00254|IGLC3*01|HOMO SAPIENS|F|C-REGION|1..312|312 NT|1| | | |104 AA|104+0=104|PARTIAL IN 5'| |
G001-04	2168	307	1081	98.1	106	11.25	EF589415|IGLC3*03|HOMO SAPIENS|C-REGION
G001-05	129	26	105	44.0	100	10.56	P01704|IGLV2-11*02|HOMO SAPIENS|V-REGION

>PtT1-λ_IGLV2-14_IGLJ3_IGLC3
QSALTQPASVAGSPGQSITLSCTGSSSDIGNYKYVSWYQQYPGKAPKLLIYDISERPSGLSSRFSGSKSGNTASLTISGLQAEDEADFYCSSYTSNNTYV
FGGGTRLTVLGQPKAAPSVTLFPPSSEELQANKATLVCLISDFYPGAVTVAWKADSSPVKAGVETTTPSKQSNNKYAASSYLSLTPEQWKSHKSYSCQVT
HEGSTVEKTVAPTECS

Supplementary Figure S10. Database-search confirmation of the assembled urinary light-chain sequences using novor.cloud. For each patient, the corresponding assembled sequence was the highest-scoring match and achieved 100% sequence coverage**.**

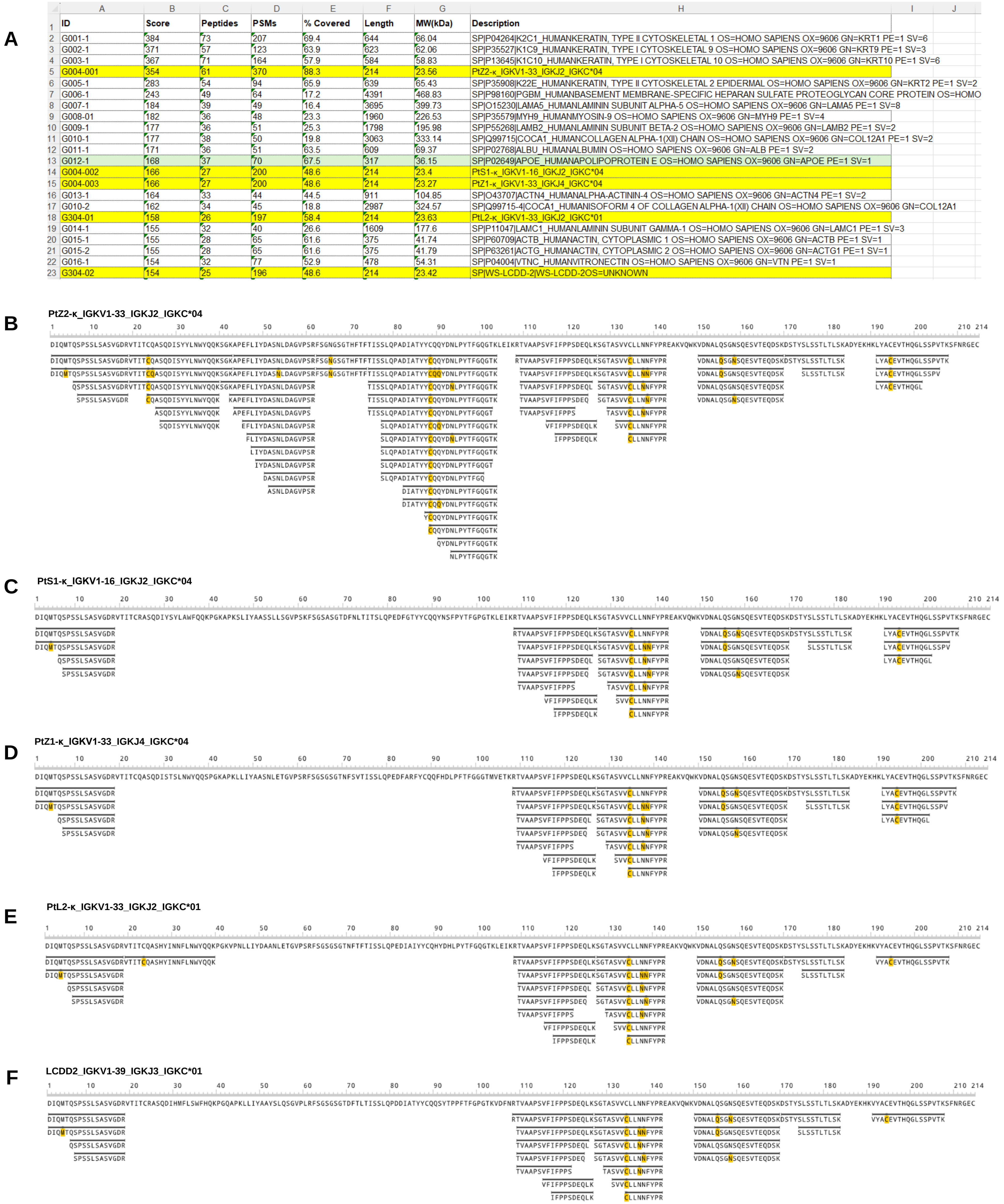

ID	Score	Peptides	PSMs	% Covered	Length	MW(kDa)	Description
G001-1	384	73	207	69.4	644	66.04	SP|P04264|K2C1_HUMANKERATIN, TYPE II CYTOSKELETAL 1 OS=HOMO SAPIENS OX=9606 GN=KRT1 PE=1 SV=6
G002-1	371	57	123	63.9	623	62.06	SP|P35527|K1C9_HUMANKERATIN, TYPE I CYTOSKELETAL 9 OS=HOMO SAPIENS OX=9606 GN=KRT9 PE=1 SV=3
G003-1	367	71	164	57.9	584	58.83	SP|P13645|K1C10_HUMANKERATIN, TYPE I CYTOSKELETAL 10 OS=HOMO SAPIENS OX=9606 GN=KRT10 PE=1 SV=6
G004-001	354	61	370	88.3	214	23.56	PtZ2-κ_IGKV1-33_IGKJ2_IGKC*04
G005-1	283	54	94	65.9	639	65.43	SP|P35908|K22E_HUMANKERATIN, TYPE II CYTOSKELETAL 2 EPIDERMAL OS=HOMO SAPIENS OX=9606 GN=KRT2 PE=1 SV=2
G006-1	243	49	64	17.2	4391	468.83	SP|P98160|PGBM_HUMANBASEMENT MEMBRANE-SPECIFIC HEPARAN SULFATE PROTEOGLYCAN CORE PROTEIN OS=HOMO
G007-1	184	39	49	16.4	3695	399.73	SP|O15230|LAMA5_HUMANLAMININ SUBUNIT ALPHA-5 OS=HOMO SAPIENS OX=9606 GN=LAMA5 PE=1 SV=8
G008-01	182	36	48	23.3	1960	226.53	SP|P35579|MYH9_HUMANMYOSIN-9 OS=HOMO SAPIENS OX=9606 GN=MYH9 PE=1 SV=4
G009-1	177	36	51	25.3	1798	195.98	SP|P55268|LAMB2_HUMANLAMININ SUBUNIT BETA-2 OS=HOMO SAPIENS OX=9606 GN=LAMB2 PE=1 SV=2
G010-1	177	38	50	19.8	3063	333.14	SP|Q99715|COCA1_HUMANCOLLAGEN ALPHA-1(XII) CHAIN OS=HOMO SAPIENS OX=9606 GN=COL12A1 PE=1 SV=2
G011-1	171	36	51	63.5	609	69.37	SP|P02768|ALBU_HUMANALBUMIN OS=HOMO SAPIENS OX=9606 GN=ALB PE=1 SV=2
G012-1	168	37	70	67.5	317	36.15	SP|P02649|APOE_HUMANAPOLIPOPROTEIN E OS=HOMO SAPIENS OX=9606 GN=APOE PE=1 SV=1
G004-002	166	27	200	48.6	214	23.4	PtS1-κ_IGKV1-16_IGKJ2_IGKC*04
G004-003	166	27	200	48.6	214	23.27	PtZ1-κ_IGKV1-33_IGKJ4_IGKC*04
G013-1	164	33	44	44.5	911	104.85	SP|O43707|ACTN4_HUMANALPHA-ACTININ-4 OS=HOMO SAPIENS OX=9606 GN=ACTN4 PE=1 SV=2
G010-2	162	34	45	18.8	2987	324.57	SP|Q99715-4|COCA1_HUMANISOFORM 4 OF COLLAGEN ALPHA-1(XII) CHAIN OS=HOMO SAPIENS OX=9606 GN=COL12A1
G304-01	158	26	197	58.4	214	23.63	PtL2-κ_IGKV1-33_IGKJ2_IGKC*01
G014-1	155	32	40	26.6	1609	177.6	SP|P11047|LAMC1_HUMANLAMININ SUBUNIT GAMMA-1 OS=HOMO SAPIENS OX=9606 GN=LAMC1 PE=1 SV=3
G015-1	155	28	65	61.6	375	41.74	SP|P60709|ACTB_HUMANACTIN, CYTOPLASMIC 1 OS=HOMO SAPIENS OX=9606 GN=ACTB PE=1 SV=1
G015-2	155	28	65	61.6	375	41.79	SP|P63261|ACTG_HUMANACTIN, CYTOPLASMIC 2 OS=HOMO SAPIENS OX=9606 GN=ACTG1 PE=1 SV=1
G016-1	154	32	77	52.9	478	54.31	SP|P04004|VTNC_HUMANVITRONECTIN OS=HOMO SAPIENS OX=9606 GN=VTN PE=1 SV=1
G304-02	154	25	196	48.6	214	23.42	SP|WS-LCDD-2|WS-LCDD-2OS=UNKNOWN

Supplementary Figure S11. Identification of the urine-derived light-chain sequence as the highest-scoring light-chain in renal amyloid deposits from patient PtZ2-κ following tryptic digestion.

(A) Protein identification results from database searching of laser microdissected Congo red-positive renal amyloid deposits following tryptic digestion. The top 22 identified proteins are shown, ranked by protein score. Immunoglobulin light-chain identifications are highlighted in yellow, while established amyloid signature proteins are highlighted in green. The urine-derived assembled light-chain sequence (PtZ2-κ_IGKV1-33_IGKJ2_IGKC*04) was the highest-scoring light-chain database match.

(B–F) Sequence coverage maps of the five highest-ranking immunoglobulin light-chain identifications in the renal amyloid proteome. The urine-derived sequence (PtZ2-κ_IGKV1-33_IGKJ2_IGKC*04) showed the highest protein score, with extensive peptide support across both the variable and constant regions. In contrast, lower-ranking light-chain identifications were supported predominantly by conserved constant-region peptides and showed little or no peptide coverage of patient-specific variable-region sequences, with the few variable-region peptides detected being confined to highly conserved framework regions.

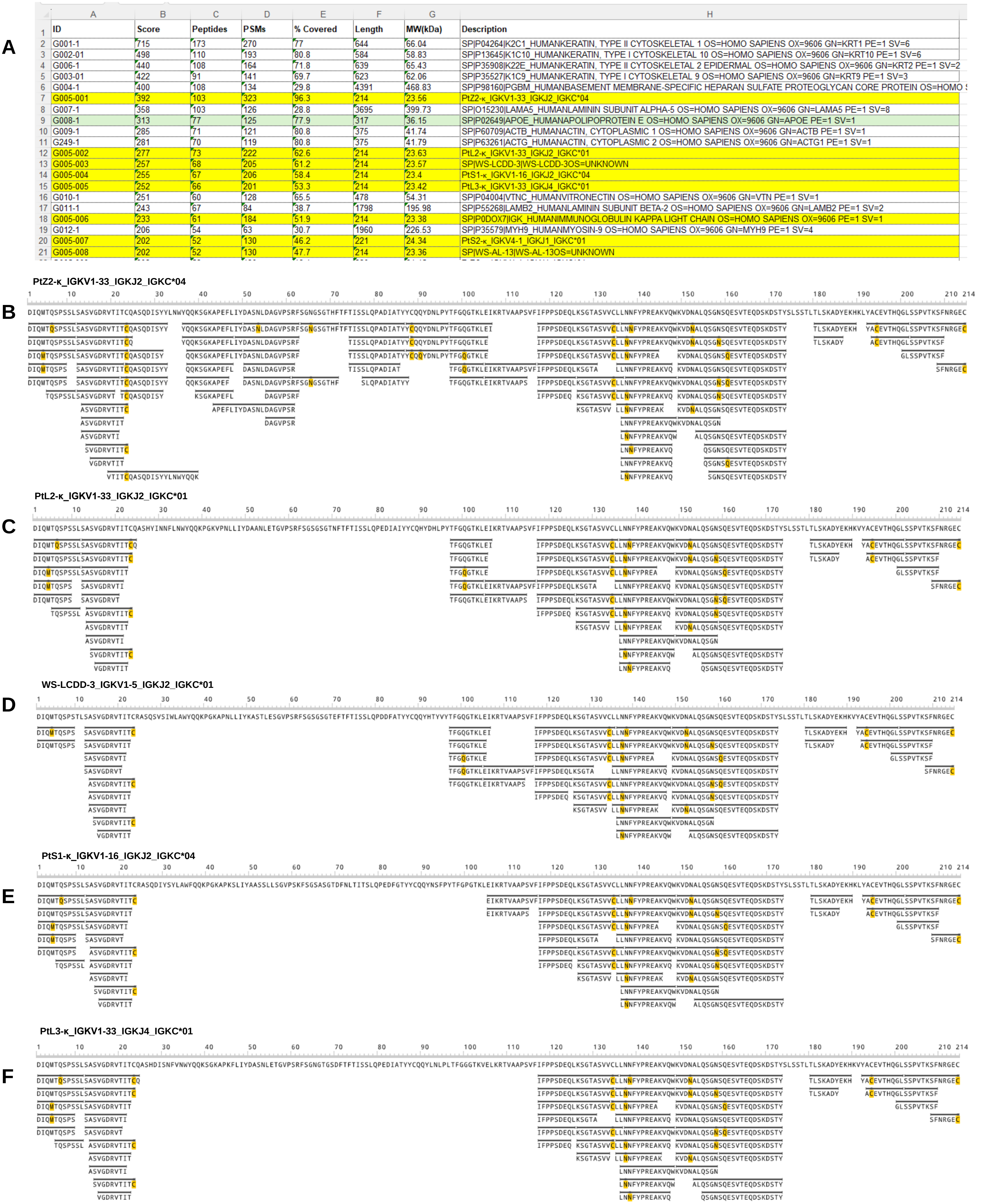

ID	Score	Peptides	PSMs	% Covered	Length	MW(kDa)	Description
G001-1	715	173	270	77	644	66.04	SP|P04264|K2C1_HUMANKERATIN, TYPE II CYTOSKELETAL 1 OS=HOMO SAPIENS OX=9606 GN=KRT1 PE=1 SV=6
G002-01	498	110	193	80.8	584	58.83	SP|P13645|K1C10_HUMANKERATIN, TYPE I CYTOSKELETAL 10 OS=HOMO SAPIENS OX=9606 GN=KRT10 PE=1 SV=6
G006-1	440	108	164	71.8	639	65.43	SP|P35908|K22E_HUMANKERATIN, TYPE II CYTOSKELETAL 2 EPIDERMAL OS=HOMO SAPIENS OX=9606 GN=KRT2 PE=1 SV=2
G003-01	422	91	141	69.7	623	62.06	SP|P35527|K1C9_HUMANKERATIN, TYPE I CYTOSKELETAL 9 OS=HOMO SAPIENS OX=9606 GN=KRT9 PE=1 SV=3
G004-1	400	108	134	29.8	4391	468.83	SP|P98160|PGBM_HUMANBASEMENT MEMBRANE-SPECIFIC HEPARAN SULFATE PROTEOGLYCAN CORE PROTEIN OS=HOMO S
G005-001	392	103	323	96.3	214	23.56	PtZ2-κ_IGKV1-33_IGKJ2_IGKC*04
G007-1	358	103	126	28.8	3695	399.73	SP|O15230|LAMA5_HUMANLAMININ SUBUNIT ALPHA-5 OS=HOMO SAPIENS OX=9606 GN=LAMA5 PE=1 SV=8
G008-1	313	77	125	77.9	317	36.15	SP|P02649|APOE_HUMANAPOLIPOPROTEIN E OS=HOMO SAPIENS OX=9606 GN=APOE PE=1 SV=1
G009-1	285	71	121	80.8	375	41.74	SP|P60709|ACTB_HUMANACTIN, CYTOPLASMIC 1 OS=HOMO SAPIENS OX=9606 GN=ACTB PE=1 SV=1
G249-1	281	70	119	80.8	375	41.79	SP|P63261|ACTG_HUMANACTIN, CYTOPLASMIC 2 OS=HOMO SAPIENS OX=9606 GN=ACTG1 PE=1 SV=1
G005-002	277	73	222	62.6	214	23.63	PtL2-κ_IGKV1-33_IGKJ2_IGKC*01
G005-003	257	68	205	61.2	214	23.57	SP|WS-LCDD-3|WS-LCDD-3OS=UNKNOWN
G005-004	255	67	206	58.4	214	23.4	PtS1-κ_IGKV1-16_IGKJ2_IGKC*04
G005-005	252	66	201	53.3	214	23.42	PtL3-κ_IGKV1-33_IGKJ4_IGKC*01
G010-1	251	60	128	65.5	478	54.31	SP|P04004|VTNC_HUMANVITRONECTIN OS=HOMO SAPIENS OX=9606 GN=VTN PE=1 SV=1
G011-1	243	67	84	38.7	1798	195.98	SP|P55268|LAMB2_HUMANLAMININ SUBUNIT BETA-2 OS=HOMO SAPIENS OX=9606 GN=LAMB2 PE=1 SV=2
G005-006	233	61	184	51.9	214	23.38	SP|P0DOX7|IGK_HUMANIMMUNOGLOBULIN KAPPA LIGHT CHAIN OS=HOMO SAPIENS OX=9606 PE=1 SV=1
G012-1	206	54	63	30.7	1960	226.53	SP|P35579|MYH9_HUMANMYOSIN-9 OS=HOMO SAPIENS OX=9606 GN=MYH9 PE=1 SV=4
G005-007	202	52	130	46.2	221	24.34	PtS2-κ_IGKV4-1_IGKJ1_IGKC*01
G005-008	202	52	130	47.7	214	23.36	SP|WS-AL-13|WS-AL-13OS=UNKNOWN

Supplementary Figure S12. Identification of the urine-derived light-chain sequence as the highest-scoring light-chain in renal amyloid deposits from patient PtZ2-κ following chymotryptic digestion.

(A) Protein identification results from database searching of laser-microdissected Congo red-positive renal amyloid deposits following chymotryptic digestion. The top 20 identified proteins are shown, ranked by protein score. Immunoglobulin light-chain identifications are highlighted in yellow, while established amyloid signature proteins are highlighted in green. The urine-derived assembled light-chain sequence (PtZ2-κ_IGKV1-33_IGKJ2_IGKC*04) was the highest-scoring light-chain database match.

(B–F) Sequence coverage maps of the five highest-ranking immunoglobulin light-chain identifications in the renal amyloid proteome. The urine-derived sequence (PtZ2-κ_IGKV1-33_IGKJ2_IGKC*04) showed the highest protein score, with extensive peptide support across both the variable and constant regions. In contrast, lower-ranking light-chain identifications were supported predominantly by conserved constant-region peptides and showed little or no peptide coverage of patient-specific variable-region sequences, with the few variable-region peptides detected being confined to highly conserved framework regions.

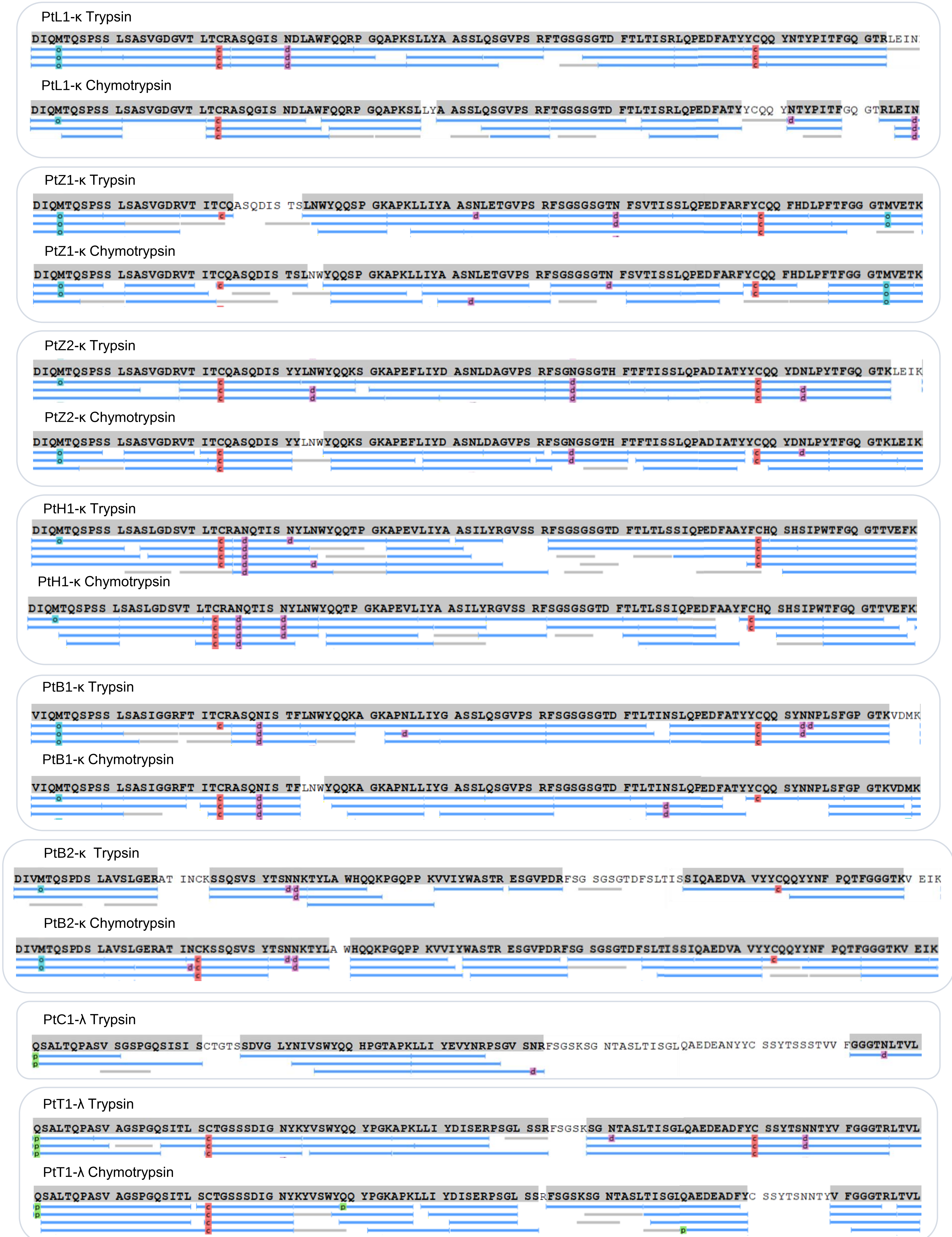

Supplementary Figure S13. PEAKS Studio visualization of V_L peptide coverage in laser-microdissected renal amyloid deposits. Patient-matched urine-assembled light-chain sequences were searched against LC–MS/MS datasets acquired from laser-microdissected renal amyloid deposits. For each patient, the trypsin- and chymotrypsin-digested datasets are shown separately, except for the PtC1-λ chymotrypsin dataset, in which the corresponding light-chain sequence was not identified by PEAKS DB. Blue bars indicate peptides identified by database searching, whereas gray bars indicate peptide sequences assigned by the *de novo* sequencing algorithm. Gray background shading of the amino acid sequence denotes V_L residues covered by identified peptides, whereas unshaded residues indicate regions without peptide coverage. The sequence-coverage maps were exported directly from PEAKS Studio, with only the upper three to five rows of peptide mappings shown for each dataset.
